# Generalized Brewster effect in dielectric metasurfaces

**DOI:** 10.1038/ncomms10362

**Published:** 2016-01-19

**Authors:** Ramón Paniagua-Domínguez, Ye Feng Yu, Andrey E. Miroshnichenko, Leonid A. Krivitsky, Yuan Hsing Fu, Vytautas Valuckas, Leonard Gonzaga, Yeow Teck Toh, Anthony Yew Seng Kay, Boris Luk'yanchuk, Arseniy I. Kuznetsov

**Affiliations:** 1 Data Storage Institute, A*STAR (Agency for Science, Technology and Research), 2 Fusionopolis Way, #08-01, Innovis 138634, Singapore; 2 Nonlinear Physics Centre, Research School of Science and Engineering, The Australian National University, Acton, Australian Capital Territory 2601, Australia; 3 Department of Electrical and Computer Engineering, National University of Singapore, 1 Engineering Drive 2, Singapore 117576, Singapore

## Abstract

Polarization is a key property defining the state of light. It was discovered by Brewster, while studying light reflected from materials at different angles. This led to the first polarizers, based on Brewster's effect. Now, one of the trends in photonics is the study of miniaturized devices exhibiting similar, or improved, functionalities compared with bulk optical elements. In this work, it is theoretically predicted that a properly designed all-dielectric metasurface exhibits a generalized Brewster's effect potentially for any angle, wavelength and polarization of choice. The effect is experimentally demonstrated for an array of silicon nanodisks at visible wavelengths. The underlying physics is related to the suppressed scattering at certain angles due to the interference between the electric and magnetic dipole resonances excited in the nanoparticles. These findings open doors for Brewster phenomenon to new applications in photonics, which are not bonded to a specific polarization or angle of incidence.

## Abstract (web summary)

Devices with greater freedom are desired in nanophotonics. Here, the authors demonstrate theoretically and experimentally that the generalized Brewster effect can be observed in an all-dielectric metasurface potentially for any angle, wavelength and polarization, due to electric and magnetic dipole interference.

## Graphical abstract

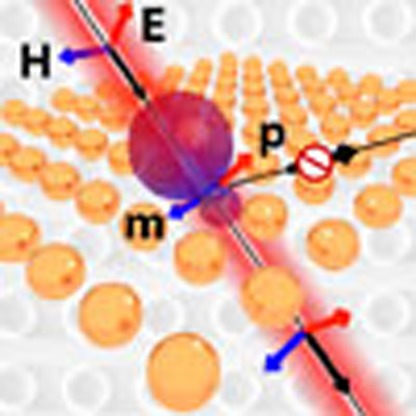

The oldest, and probably simplest, way to obtain linearly polarized light starting from unpolarized one is impinging it on a dielectric interface at the so-called Brewster's angle. In this way, the reflected light will only have electric field component parallel to the interface. Well-understood since the 1820s after the pioneering work of Fresnel, and experimentally known since the early 1810s from works of Malus and Brewster^1^ (see also ref. 2 for a succinct historical perspective), the Brewster's angle for homogeneous isotropic non-magnetic media can be defined as the angle for which Fresnel's reflection coefficient for *p*-polarized light (that is, with the electric field parallel to the plane of incidence) vanishes, 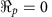. An alternative definition states that Brewster's angle is the one at which the reflected and refracted waves are orthogonal, thus fulfilling the condition *θ*_*i*_+*θ*_*t*_=*π*/2, where *θ*_*i*_ is the angle of incidence and *θ*_*t*_ is the angle of refraction/transmission. The common microscopic interpretation of this effect is illustrated in Fig. 1a. In response to the driving electromagnetic wave, electric dipoles are induced within the material. These dipoles oscillate along the direction of the electric field, that is, perpendicular to the propagation direction. As the far-field power radiated by a dipole vanishes along its oscillation axis, whenever the dipoles and the reflection direction are parallel, no radiation is emitted into that direction and reflection is inhibited. In all other directions apart from that of refraction, radiation is compensated by the rest of the dipoles within the medium. If polarization is switched, as shown in Fig. 1b, due to the non-zero radiation in the plane perpendicular to the dipole, it is clear that such effect cannot be achieved.

The situation becomes more interesting when one considers a material that has both electric and magnetic dipoles excited in response to the electric and magnetic components of the incident wave. Such materials should have both electric permittivity and magnetic permeability different from unity (*ɛ*≠1, *μ*≠1). In this case, the radiation pattern is no longer zero in the direction of oscillation of any of the orthogonal electric or magnetic dipoles (due to the non-zero contribution of the orthogonal dipole) and thus the classical Brewster effect can no longer be observed. Instead, there can be other particular directions at which the collective radiation of both dipoles vanishes due to their destructive interference, as predicted by Kerker *et al.*^3^ in early 80s. These directions are determined by the relative amplitudes and phases of the dipoles. In the macroscopic picture, this interference may lead to the appearance of an analogue to Brewster's angle defined by both electric and magnetic properties of the material, as depicted in Fig. 1c. This is, the ratio *ɛ*/*μ* determines the angle at which the condition 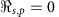 is satisfied^4^. More importantly, inhibition of radiation from a pair of dipoles can happen at any angle and in any of the two oscillation planes depending on their relative amplitudes and phases. Thus, for such a material Brewster's angle may exist, potentially, for any of the two polarizations and at any angle of incidence (even below 45° without leading to total internal reflection at some higher angles). Both polarizations cannot, however, simultaneously have zero reflection for a given angle, except for the very particular case of *ɛ*=*μ* (impedance matched) at normal incidence^5^. In this case, each polarizable portion of matter will have induced electric and magnetic dipoles having the same amplitude and phase leading to inhibition of backscattered radiation, that is, fulfilling the so-called first Kerker's condition, originally derived for small magnetic particles^3^. In case of purely magnetic media, *μ*≠1 and *ɛ*≈1, one can find a situation when the analogue to Brewster's angle appears for *s*-polarized light, having the magnetic field vector parallel to the plane of incidence, which is orthogonal to the conventional Brewster effect in dielectric media.

All this findings remained a mere theoretical curiosity for almost 20 years, since for natural materials the magnetic response is typically very weak at optical frequencies (*μ*≈1). Nevertheless, since the advent of metamaterials new ways to produce optical magnetic response have been explored^6^,^7^,^8^. As a result some attempts have been done towards finding Brewster's angle in *s*-polarization in bulk magnetic metamaterials, both in microwaves^9^ in arrays of split ring resonators and at optical frequencies in strongly anisotropic media^10^. Recently, polarization rotation in reflection from meta-films of bi-anisotropic split rings has been theoretically studied at microwave frequencies in connection to Brewster effect^11^.

In this paper, it is demonstrated both theoretically and experimentally that the generalized Brewster effect can be observed, potentially, in any two-dimensional (2D) sub-diffractive arrangement of high-index dielectric nanoparticles, or in any other system where strong electric and magnetic resonances can be efficiently excited. It is shown that this effect is a direct consequence of the angle-suppressed radiation/scattering due to interference between the electric and magnetic dipoles excited in the particles within the array, thus connecting two apparently unrelated phenomena such as Brewster's angle and general Kerker's conditions. Silicon (Si) nanoparticles are specifically considered, for which such resonances have been broadly studied both theoretically^12^,^13^,^14^ and experimentally^15^,^16^,^17^. They have attracted particular attention within the field of artificial magnetism at optical frequencies^12^,^13^,^14^,^15^,^16^,^17^ due to their low intrinsic losses and CMOS compatibility, which holds promise for finding real world applications. Their exciting properties regarding magnetic near-field enhancement^18^,^19^,^20^,^21^,^22^ and directional scattering^23^,^24^,^25^,^26^,^27^,^28^, together with their low dissipation, makes them ideal nanoantennas for visible and near-infrared light^29^. The possibility to realize the first Kerker's condition^23^,^24^,^25^,^26^,^27^ has also inspired studies on using them as ideal Huygens' sources in highly efficient transmissive metasurfaces^30^,^31^,^32^. Also their strong interaction with light, leading to high reflection and phase accumulation, makes them ideal candidates to act as efficient reflectors or phase-controlled mirrors^12^,^33^,^34^,^35^. The present study comes to extend this already broad realm with new fascinating properties. Moreover, novel generalized Brewster phenomenon giving great degree of freedom in polarization and incident angles may open doors to multiple new applications in photonics, which could not be achieved with standard Brewster effect in conventional dielectric media.

## Results

### Generalized Brewster effect in arrays of silicon spheres

**Figure 1:**
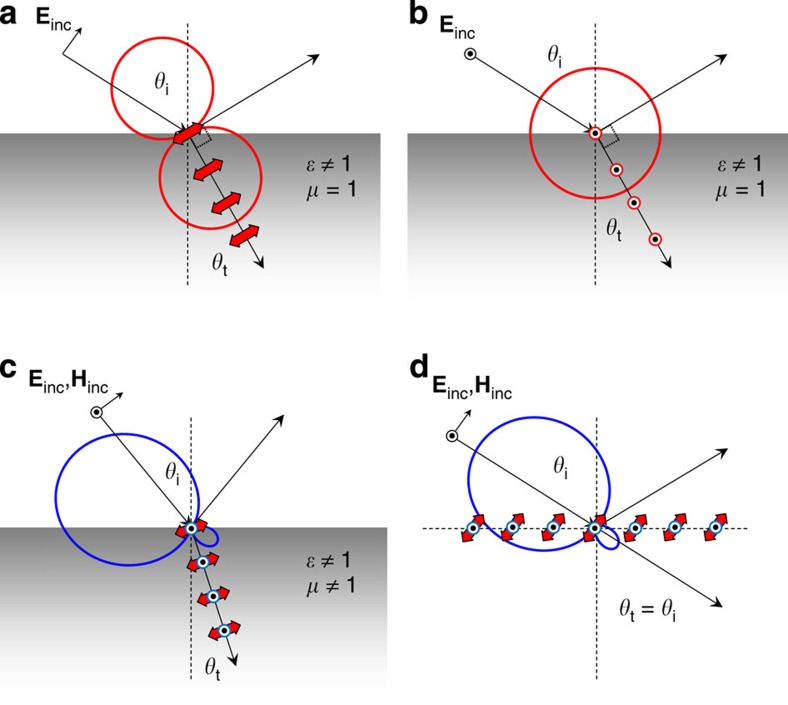
Microscopic interpretation of Brewster effect and proposed metasurface. (**a**) *p*-polarized light impinging on a dielectric medium, *ɛ*≠1 and *μ*=1, under usual Brewster's condition. Red line shows 2D emission diagram of electric dipoles excited inside the material by the refracted wave. (**b**) Same as in **a** but for *s*-polarized incidence, for which no Brewster effect can be observed. (**c**) Generalized Brewster effect for a dielectric medium with electric and magnetic response, *ɛ*≠1 and *μ*≠1. Blue line shows 2D emission diagram of interfering electric and magnetic dipoles excited inside the material by the refracted wave. (**d**) Generalized Brewster effect in the proposed array of high refractive index nanoparticles.

**Figure 2:**
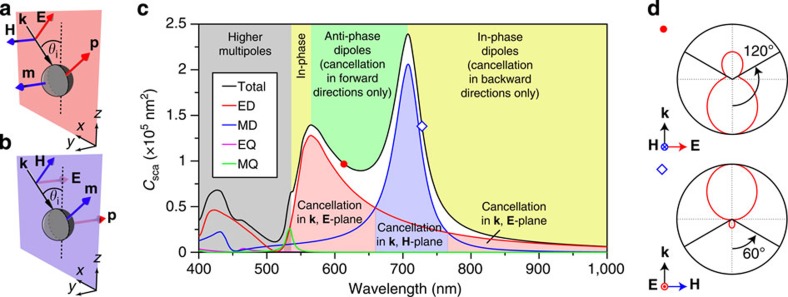
Optical properties of a silicon spheres in air under plane wave illumination. (**a**,**b**) Schematic representation of the two situations studied. (**c**) Total scattering cross section (black curve) and contributions from the electric dipole (red curve), magnetic dipole (blue curve), electric quadrupole (magenta curve) and magnetic quadrupole (green curve) from a silicon sphere with diameter *D*=180 nm. The different shaded regions indicate those wavelength windows for which the induced electric and magnetic dipoles are approximately in phase (yellow) or in anti-phase (green), and those for which the electric dipole (light red) or the magnetic dipole (light blue) dominate over the other. (**d**) Far-field radiation patterns for two wavelengths leading to inhibition of radiation at 60° with respect to the forward- (*λ*_1_=614 nm, red solid circle) and backward- (*λ*_2_=728 nm, blue hollow diamond) scattering directions. In the first case zero radiation is only possible in the plane parallel to the electric field whereas in the second it is only possible in the perpendicular one.

This section aims to show that the important phenomenology associated to the generalized Brewster effect (see Supplementary Note 1 and associated Supplementary Figs 1, 2 and 3 for a concise review of it) can be found in sub-diffractive, 2D arrays of silicon spheres. We will demonstrate that, in a similar way as in continuous medium with both electric and magnetic response, the suppression of reflection originates from the interference between electric and magnetic dipoles induced in the particles in the array, as schematically depicted in Fig. 1d. For this purpose, let us first start considering a single silicon nanosphere under plane wave illumination (Fig. 2a,b), for which the required electric and magnetic dipole modes can be efficiently excited. The scattering cross section (*C*_sca_) for a sphere with diameter *D*=180 nm, as computed analytically with Mie theory^36^, is depicted in Fig. 2c. Partial scattering cross-sections by the first excited resonant modes, namely the electric and magnetic dipoles and the electric and magnetic quadrupoles are also shown. As can be seen, the usual hierarchy of resonances in high-contrast dielectric nanoparticles starts with the lowest-energy magnetic dipole followed by the electric dipole mode^12^,^13^,^14^,^15^,^16^. Thus, whenever higher-order modes are negligible each sphere can be accurately described by a pair of these dipoles.

Kerker *et al.*^3^ showed that, in such systems, the scattered far-field can be completely polarized parallel or perpendicular to the scattering plane in some particular observation direction, and this direction depends on the relative strength of the induced electric (**p**) and magnetic (**m**) dipoles. Originally derived for magnetic spheres, this result relates to interference in the electric far-field radiated by a pair of such dipoles^37^, which reads:


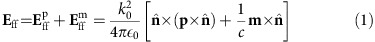


with *k*_0_=2*π*/*λ* the wavenumber and 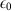 and *c* the permittivity and speed of light in vacuum, respectively, and 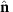 the unit vector in the observation direction.

Consider now the particular situations depicted in Fig. 2a,b. It also follows from (1), see Supplementary Note 2, that in the plane containing the incident wave-vector and the induced electric dipole (highlighted in Fig. 2a), the radiated electric field vanishes in the observation direction defined by the angle *θ* if:


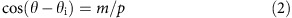


in which *p* and *m* are the complex amplitudes of electric and magnetic dipoles. In the orthogonal plane, which contains the incident wave-vector and the induced magnetic dipole (case depicted in Fig. 2b), the field vanishes when:


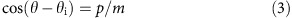


Note that the backscattering direction is defined by *θ*=*θ*_i_. In this direction, the field vanishes when *p*=*m* (first Kerker's condition^3^). Note also that equations (2) and (3) are, in general, complex and become real only when the dipoles are in phase or anti-phase. From these equations it can be seen that radiation can be totally suppressed for angles in backward directions |*θ*−*θ*_i_|≤*π*/2 exclusively if the dipoles are in phase (*p* and *m* having the same sign), and in forward directions (|*θ*−*θ*_i_|≥*π*/2) if they are in anti-phase (*p* and *m* having opposite sign). The spectral regions in which the induced dipoles are approximately in phase or anti-phase for the silicon sphere are highlighted in Fig. 2c by yellow and green shading colours, respectively. They indicate the spectral ranges for which scattering cancellation in forward and backward directions may happen.

The partial scattering cross-sections^36^ associated with the electric 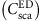
and magnetic 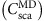
dipoles are proportional to the squared modulus of the dipole moments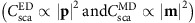, and this allows to recast equations (2) and (3) as:


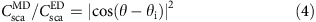



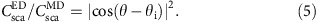


It immediately follows from (4) that the electric dipole scattering must dominate 
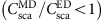
 to achieve cancellation in the plane containing the electric dipole. Similarly, it follows from (5) that the magnetic dipole scattering should be dominant 
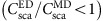
 to achieve the scattering cancellation in the plane containing the magnetic dipole. The regions of dominant electric and magnetic dipoles are highlighted in Fig. 2c by red and purple shading colours, respectively.

In Fig. 2d the 2D scattering pattern of the Si sphere computed from Mie theory is plotted for two selected wavelengths, *λ*_1_=614 nm and *λ*_2_=728 nm, in the plane containing the incident wave-vector and the electric or magnetic dipole, respectively. Vanishing scattering intensity angles predicted by equations (4) and (5), respectively, are also shown. At *λ*_1_ the electric dipole dominates and the dipoles are in anti-phase leading to scattering cancellation at an angle |*θ*−*θ*_i_|≥*π*/2 in the plane containing the incident electric field.

At *λ*_2_ the magnetic dipole contribution is dominant and dipoles are in phase leading to scattering cancellation at an angle |*θ*−*θ*_i_|≤*π*/2 in the plane containing the incident magnetic field. Relative amplitudes and phases are such that interference suppresses radiation at 60° with respect to the forward- and back-scattering directions, respectively.

**Figure 3:**
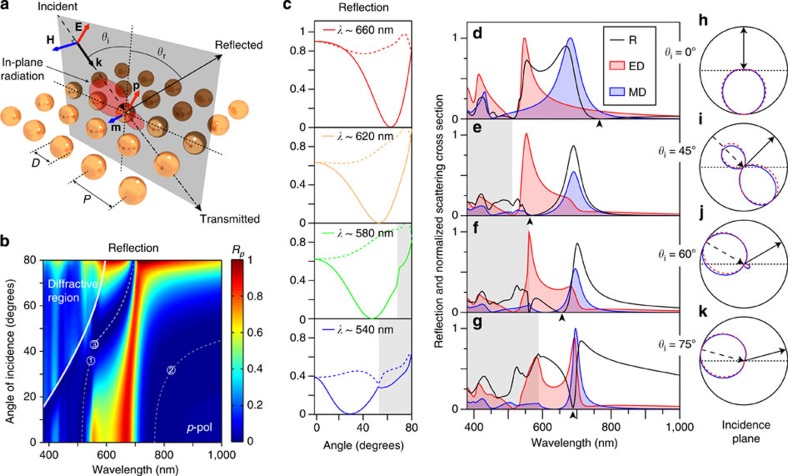
Simulated optical response of arrays of silicon spheres under *p*-polarized oblique incidence. (**a**) Scheme of the simulated system. (**b**) Numerically calculated reflection versus wavelength and angle of incidence for a square lattice of silicon spheres with diameter *D*=180 nm and pitch *P*=300 nm. The diffractive region is indicated and delimited by a thick, solid, white line. The light-white, dashed lines are guides to the eye to help identifying the low reflectivity regions of interest. (**c**) Reflection versus angle of incidence for selected wavelengths showing the strong dependence of Brewster's angle on wavelength as well as the possibility of achieving values below 45°. Solid lines correspond to *p*-polarization while dashed lines are the corresponding curves for *s*-polarization. Grey-shaded areas mark the spectral regions affected by diffraction. (**d**,**g**) Reflection (black curve), together with electric dipole (red curve and corresponding shaded area) and magnetic dipole (blue curve and corresponding shaded area) contributions to scattering (normalized to their common maximum) as a function of wavelength for the cases of normal incidence and oblique incidence with *θ*_i_=45, 60 and 70°, respectively. Diffractive region is indicated by a shaded grey area. (**h**–**k**) Radiation patterns in the plane of incidence numerically computed via Stratton–Chu formulas (blue solid curve) and from the induced electric and magnetic dipoles only (red dashed curve) at the wavelength of minimum reflection (arrow heads in **d**–**g** respectively). Incidence and reflection direction are shown by arrows.

Let us now consider the case of similar spheres arranged in an infinite 2D sub-diffractive array in the *xy*-plane (Fig. 3a) under plane wave oblique incidence. It is clear that for *p*-polarized light the plane of incidence coincides with the plane that contains the incident electric field and the induced electric dipoles (as in Fig. 2a). Correspondingly, for *s*-polarization the incidence plane contains the magnetic field and the induced magnetic dipoles (as in Fig. 2b). Although the effective dipoles induced in the particles in the array are different from the single particle case owing to the lattice interactions^38^, they still radiate according to equations (1)–(3), 6, 7 in the plane of incidence. Note that interference from different sites in the infinite array makes radiation of the whole system allowed only as plane waves along the diffraction directions. In the case of sub-diffractive arrays, this implies radiation in the reflection and transmission directions only. Therefore, if the induced dipoles do not radiate along the direction of reflection, no reflection at all will occur in the system, leading to the Brewster's condition (see Supplementary Note 3, and the associated Supplementary Fig. 4, for a demonstration in the context of phased arrays). Following the discussion above, in such systems this may happen for both *s*- and *p*-polarized incident waves.

In the following, we consider an infinite square lattice of silicon spheres with diameter *D*=180 nm and period *P*=300 nm, as depicted in Fig. 3a, and study its reflection properties as a function of the wavelength and angle of incidence. We start with *p*-polarized light. Simulated results obtained by means of finite element method (see Methods section for details) are shown in Fig. 3b. At normal incidence, the electric and magnetic resonances of the particles lead to the appearance of well-known bands of high reflectivity^12^,^33^,^34^. However, oblique incidence strongly changes this behaviour. At high angles of incidence one can observe three regions of extremely low reflection. Light-white, dashed lines, with numbering ranging from 1 to 3 are included in the figure as guides to the eye to ease their location and referencing. The first one is a narrow region located at the blue side of the resonances (∼515 nm). It is present at normal incidence and slightly redshifts for increasing angles (from 0 up to ∼40°). The second one, located in the red side of the resonances (∼790 nm), is also present at normal incidence and strongly redshifts with increasing angles. Finally, a broad region, both in bandwidth (∼150 nm) and angles of incidence (from ∼40 to ∼80°), appears at higher angles, spectrally located between the positions of the electric and magnetic dipole resonances observed at normal incidence. Importantly, the angle of minimum reflection strongly depends on the wavelength and varies in the wide range. Figure 3c shows the angular dependence of reflection at some selected wavelengths to better illustrate this effect. One can observe that reflection of *p*-polarized light (solid lines) turns into zero at some particular angle of incidence, resembling the conventional Brewster effect in dielectric media, while no special features are observed for *s*-polarized light at this angle (corresponding dashed lines). However, there are two major peculiarities of this system, which should be highlighted. First, the range of angles at which the reflection minimum is observed covers almost the whole 0–90° span, not being restricted to angles above 45° (opposite to the conventional Brewster effect). Second, as will be shown next, the effect is not restricted to *p*-polarization, thus gathering the main features of generalized Brewster phenomenon. It is important to mention that this effect is not related to diffraction. The first non-zero diffraction order, indicated as a dashed white line in Fig. 3b and as shaded regions in Fig. 3c, appears out of the range of wavelengths and angles for which the effect is observed.

Let us now focus on the spectral region between the electric and magnetic dipole resonances. In Fig. 3d–g the case of normal incidence is shown, together with some cases with zero reflection in that region, namely 45, 60 and 75° incidence. For normal incidence both reflection maxima spectrally coincide with the excitation of dipolar resonances inside the particles. Zero reflection is observed at 775 nm, where the induced electric and magnetic dipoles have the same amplitudes and phases meeting the first Kerker's condition^3^,^23^,^24^,^25^,^26^,^27^, and at 515 nm, which is close to the Kerker's condition but also affected by higher-order contributions. In the cases of oblique incidence, zeros in reflection are observed at 566 nm for 45°, 657 nm for 60°, and 686 nm for 75° (indicated by arrow heads in Fig. 3d–g) showing the strong wavelength dependence.

One explanation of the emergence of the reflection minimum at higher angles could be associated with disappearance of the dipolar resonances at off-normal incidence. However, this is not the case. Both dipolar modes are still efficiently excited, and it is their mutual interference which results in the radiation inhibition in the reflection direction. Figure 3d–g show by red and blue curves (and corresponding shaded areas), the electric dipole and magnetic dipole contributions to the total scattering cross-section (*C*_sca_) from each single particle in the array, computed using the multipole decomposition technique, as explained in Methods section. As readily seen, both dipole modes are present for those angles and wavelengths for which the reflection vanishes. The dipolar contributions are dominant and higher-order modes only appear at shorter wavelengths (the complete map can be found in Supplementary Fig. 5, described in the Supplementary Note 4). Interestingly, multipole decomposition reveals that the electric dipole dominates in all the above cases. This is expected from equation (4) to be able to cancel radiation in the plane containing the electric field, which in *p*-polarized case coincides with the plain of incidence. In a simplified case with no interaction between the particles in the array, the induced dipoles should oscillate parallel to the incident field. In that case, to cancel scattering at the reflection angle *θ*=*θ*_r_=−*θ*_i_, equation (4) imposes 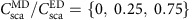 for *θ*_i_={45, 60, 75} degrees, respectively. Thus, usual Brewster at 45° is covered in this description and requires vanishing of magnetic dipole for *p*-polarization, as expected. Actual values retrieved from simulations, in which interparticle interaction is taken into account, become 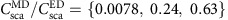, which are quite close to the interaction-free case. The second zero in reflection observed at 560 nm for *θ*_i_=60° is related to the onset of the diffractive regime (indicated as grey-shaded areas).

As a test of consistency, far-field radiation patterns from each single particle in the infinite array were computed using Stratton–Chu formulas^5^ from the fields on the surface of the sphere and plotted in the plane of incidence in Fig. 3h–k (blue solid lines). Also shown (red dashed lines) are the patterns radiated by the pair of electric **p** and magnetic **m** dipoles given by the multipole decomposition, computed through equation (1). Both patterns closely coincide and show zero radiation in the direction of the reflected wave (indicated, together with the incident one by arrows), thus confirming the dipole interference origin of the vanishing reflection regions.

**Figure 4:**
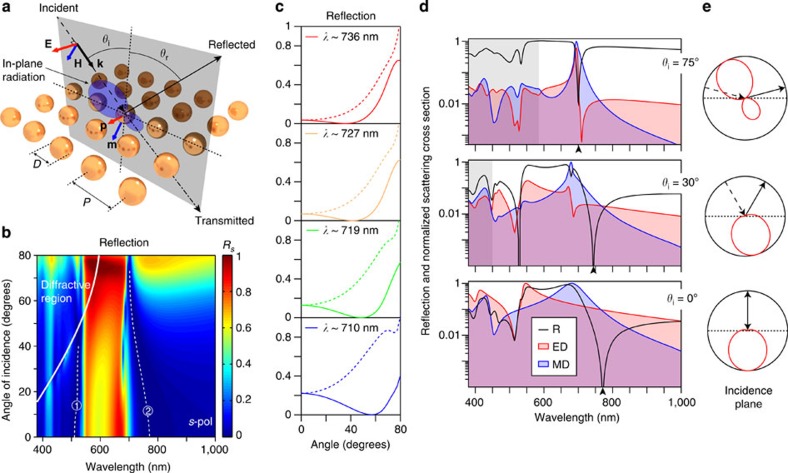
Simulated optical response of arrays of silicon spheres under *s*-polarized oblique incidence. (**a**) Scheme of the simulated system. (**b**) Numerically calculated reflection versus wavelength and angle of incidence for a square array of silicon spheres with diameter *D*=180 nm and pitch *P*=300 nm. The region for which diffraction appears is indicated and delimited by the thick, solid white line. The white dashed lines are guides to the eye to help identifying the low reflectivity regions of interest. (**c**) Reflection versus angle of incidence for selected wavelengths showing Brewster's angle for *s*-polarization. Solid lines represent *s*-polarization while dashed lines are the corresponding curves for *p*-polarization. Dependence on wavelength and the possibility to achieve values below 45° are observed. (**d**) Reflection (black curve) and electric dipole (red curve and corresponding shaded area, electric dipole (ED)) and magnetic dipole (blue curve and corresponding shaded area, magnetic dipole (MD)) contributions to the scattering (normalized to their common maximum) from a single sphere in the array for several angles of incidence. Log_10_ scale is used for better visualization of the minima. Diffractive regions are indicated by the shaded grey area. (**e**) Associated radiation patterns of each single particle in the array in the plane of incidence at the wavelengths of minimum reflection (arrow heads in **d**).

Let us now switch to the case of *s*-polarized incidence (as depicted in Fig. 4a) to show that similar effects can be obtained. The change in polarization makes the plane of incidence coincide with that containing the magnetic field in the analysis for a single sphere, thus obeying equations (3) and (5). The simulated reflection versus wavelength and angle of incidence for the same array of spheres in *s*-polarized case is shown in Fig. 4b. Two narrow band frequency windows of vanishing reflection, shifting very weakly with the angle of incidence, can be observed starting at ∼515 and 770 nm for normal incidence, indicated by the light-white, dashed lines with numbers 1 and 2, respectively. Also, an omni-directional, high-reflectivity region is observed in between, analogous to that reported for high-index infinite cylinders^39^. Brewster effect in this polarization is evidenced by plotting, as in Fig. 4c, the reflection against the angle of incidence for several wavelengths. We focus on the narrow band 2, observed between 700 and 750 nm, for which no higher-order multipoles are present. For *s*-polarized light (shown as solid lines) emergence of Brewster's angle is apparent, while no special features are observed for *p*-polarization (dashed lines). As readily observed, strong dependence on wavelength and span over the whole 0–80° simulated range are also observed for *s*-polarization.

Now we show that the origin of Brewster's angle in this polarization is totally analogous to that of *p*-polarization. To this end, particular angles are plotted in Fig. 4d together with the electric dipole and magnetic dipole partial scattering cross-sections (normalized to their common maximum). For normal incidence spectral position of the dip corresponds to the first Kerker's condition at which electric and magnetic dipoles have similar amplitude and phases^3^,^23^,^24^,^25^,^26^,^27^. The observed weak blue-shift of this dip with increased angle of incidence is a consequence of the particular shape of the resonances excited in the particles and their mutual interplay, which allows fulfilling equation (5) for every angle in a narrow spectral region. Note that within the whole range of wavelengths and angles with vanishing reflection, the magnetic dipole contribution is higher than the electric dipole one, as predicted by equation (5). Similar to the case of *p*-polarized incidence, the radiation patterns of each single particle in the array associated with zero-reflection wavelengths show no radiation in the reflection direction, thus confirming the interference origin of the effect also in *s*-polarization as depicted in Fig. 4e.

It is important to stress that the observed spectral and angular behaviour of the zero-reflection regions in the metasurface (Figs 3b and 4b) can be directly related to the scattering properties of the single building-blocks through amplitudes and phases of the induced dipoles, as described in detail in Supplementary Note 5 (and associated Supplementary Figs 6 and 7). Thus, engineering these parameters, for example, through the geometry of the inclusions, could lead to the generalized Brewster effect, potentially, at any desired angle, frequency and polarization of interest.

### Experimental verification with arrays of silicon nanodisks

**Figure 5:**
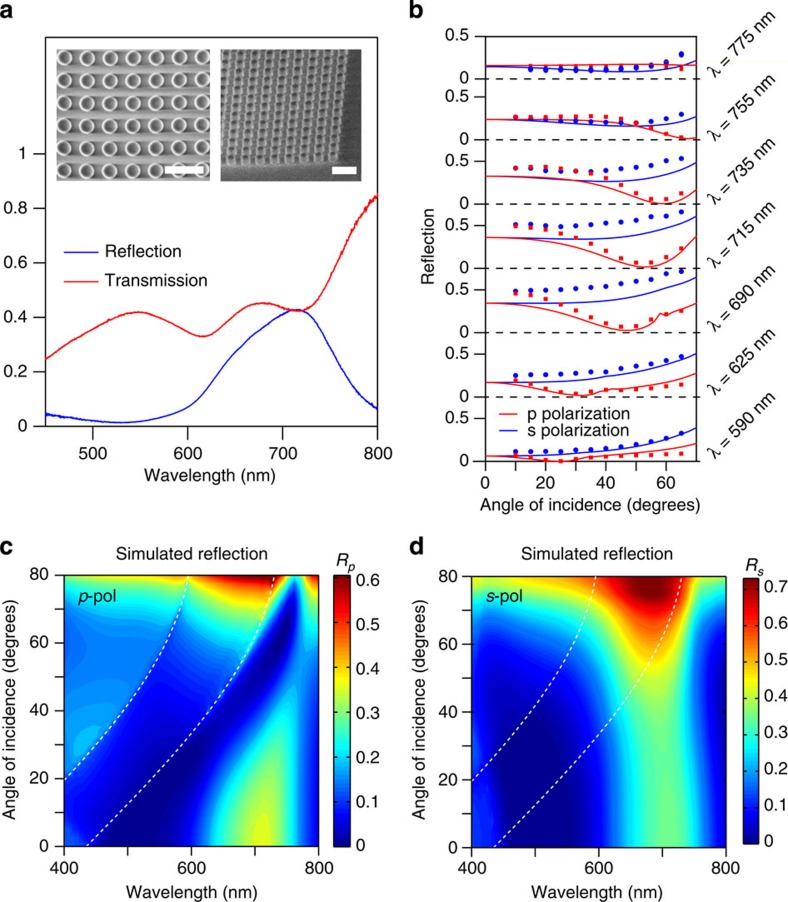
Angular reflection of light from arrays of silicon nanodisks over a glass substrate. (**a**) Experimentally measured reflection (blue solid curve) and transmission (red solid curve) of a square lattice of silicon disks with diameter *D*=180 nm, height *H*=150 nm and pitch *P*=300 nm under normal incidence. The insets show the top (left) and tilted (right) SEM images of the measured sample. Scale bar, 500 nm. (**b**) Reflection versus angle of incidence measured for different wavelengths under *p*-polarized (red circles) and *s*-polarized (blue circles) illumination. The corresponding simulated data, obtained for *D*=170 nm and *H*=160 nm with the same pitch are shown as solid curves. (**c**,**d**) Maps of simulated values of reflection as a function of angle of incidence and wavelength for *p*-polarized and *s*-polarized incidence, respectively. White dashed lines indicate the onset of the different diffraction orders.

To experimentally demonstrate the generalized Brewster effect, an array of silicon nanodisks was fabricated on a fused silica substrate (as described in Methods section) through silicon film deposition, electron-beam lithography and etching. Disks are chosen for ease of fabrication and, for aspect ratios close to unity, they are expected to have similar optical properties to spheres. The actual diameter is around *D*=180 nm, height *H*=150 nm and array pitch *P*=300 nm (see SEM images of the fabricated array in the insets to Fig. 5a). Angular-dependent reflection measurements were performed using a home-built free-space microscopy set-up (see Methods section for details). The measured reflection and transmission spectra under normal incidence are plotted in Fig. 5a as blue and red lines, respectively.

Reflection measurements as a function of the angle of incidence for several wavelengths in the spectral region covering both electric and magnetic dipole resonances are presented as solid circles in Fig. 5b for *p*- (red) and *s*-polarized light (blue), together with results of numerical simulations (corresponding solid lines). The best agreement with the experiment was achieved for simulated diameter *D*=170 nm, height *H*=160 nm, pitch *P*=300 nm and substrate refractive index of 1.45. The origin of the small discrepancy between the experiment and simulations is due to a difference between the refractive index of the fabricated silicon and the tabulated data for α-silicon^40^ used in the simulations, suggesting that the fabricated silicon has less dissipation than that commonly found in literature (see Supplementary Note 6 for details). For *p*-polarization, it is clearly observed the appearance of a zero-reflection angle showing strong wavelength dependence and ranging from ∼25 to nearly 70° in the studied frequency range, that is, going well below 45°. For those values below 45° no sign of total internal reflection is found. These results are strongly different from conventional Brewster's angle behaviour and represent the first experimental demonstration of the generalized Brewster effect in arrays of particles with both electric and magnetic responses. Numerical simulations, shown as solid lines in Fig. 5c, closely reproduce the experimental values and demonstrate excellent agreement in the position of the minima. The slight differences in the reflection intensity, as mentioned, are due to the smaller absorption of the deposited silicon compared with the common amorphous silicon data from literature used in simulations^40^. Taking in simulations slightly lower values of the imaginary part of refractive index than in Palik^40^ (as given in Supplementary Table 1) leads to almost perfect agreement to the experiment (Supplementary Fig. 8).

To complete the picture, the full simulated maps of reflection of *p*- and *s*-polarized light as a function of angle of incidence and wavelength are shown in Fig. 5c,d. Although with some differences, the general trend observed in the simulated region strongly resembles that shown in Figs 3b and 4a for spheres. For *p*-polarized light, the minimum in reflection strongly varies both with wavelength and angle of incidence, starting in the blue side of the resonances and moving into the region between them for increasing angles. As in the case of spheres both electric dipole and magnetic dipole modes retrieved from multipole decomposition for single disk in the array are strongly excited in the regions of zero reflection (see Supplementary Note 7 and Supplementary Fig. 9). Radiation patterns of these interfering dipoles computed for two of the zero-reflection cases in *p*-polarization are shown in Supplementary Fig. 10 and discussed in Supplementary Note 8. They demonstrate vanishing intensities of the radiation in the direction of the reflected wave at the operation wavelength, thus confirming the interference origin of the observed effect.

For *s*-polarization a shallow minimum in reflection at 735, 755 and 775 nm can be observed both in simulations and experiment (Fig. 5b). These minima correspond to the tail of the vanishing reflection region (Fig. 5d) and provide further experimental evidence of the generalized Brewster effect. Discussion of the experimental plot focused on the cases of 755 and 775 nm can be found in Supplementary Fig. 9, clearly showing a minimum in reflection for angles below and above 45°. It is worth mentioning that for this particular system the complete vanishing of reflection under *s*-polarized incidence can be obtained in the spectral region ∼850 nm. However, at these wavelengths the array has very low reflectivity even at normal incidence.

Remarkably, even for the realistic system described above the generalized Brewster effect is very robust and can easily be detected in experiment, the only true requirement being the efficient excitation of electric and magnetic dipole resonances in the particles forming a sub-diffractive array.

## Discussion

According to the results shown, sub-diffractive arrays of high-permittivity dielectric nanoparticles supporting both electric and magnetic dipole resonances present a form of generalized Brewster effect leading to vanishing reflection at particular wavelengths and angles both under *p*- and *s*-polarized incidence. The phenomenon can be explained in terms of radiation interference between the electric and magnetic dipoles induced in each particle in the array and connects the angle-suppressed scattering from magneto-electric particles (usually studied in relation to first Kerker's condition) with the zero-reflection (Brewster effect) observed in 2D arrangements of such particles. As a consequence of this interference the range of zero-reflection angles spans almost over the entire 0–90° without implying total internal reflection. It shows a strong dependence on the incident wavelength and is present for both *p* and *s* polarizations. The effect has been experimentally demonstrated in dense arrays of silicon disks over a fused silica substrate, with measured zero-reflection angles ranging from 20 to 70° for wavelengths varying from 590 to 775 nm in the visible spectrum. These results represent the first experimental demonstration of the generalized Brewster's effect at optical frequencies in particle arrays with both electric and magnetic response to incident light.

Since this effect is a universal phenomenon related to the directional interference of resonances excited in the particles, it is foreseen that it will be observed in a variety of systems, provided they present electric and magnetic responses. Moreover, tuning the shape and material properties of the particles may lead to almost-on-demand Brewster's effect with regard to polarization, wavelength and angle of incidence. Taking advantage of the strongly resonant character of the structures may bring opportunities for design of efficient sub-wavelength-thick polarizers with a great degree of freedom.

## Methods

### Numerical simulations

Finite element method was used to compute the reflection, transmission and absorption of light from infinite square arrays of silicon spheres (commercial COMSOL Multiphysics software was used). Experimentally measured values of the refractive index of crystalline silicon, taken from ref. 40, were used in the simulations. The simulation domain consisted of a single unit cell with Bloch boundary conditions applied in the periodicity directions (*x* and *y* axes) to simulate an infinite lattice. The so-called scattered field formulation of the problem was used. The exciting field was defined as a plane wave with the electric field in the incidence plane for *p*-polarized light and perpendicular to it for *s*-polarized light. Perfectly matched layers were applied in the top and bottom directions to absorb all scattered fields from the system. Additionally two planes, Σ_±_, perpendicular to the *z* axis at *z*=±450 nm were used as monitors to compute the reflected and transmitted power. Reflection was computed as the flux of the Poynting vector of the scattered fields in the Σ_−_ plane normalized to the power of the plane wave in the same area. Total fields instead of scattered ones were considered in Σ_+_ to compute transmission. Absorption was computed as the volume integration of the Ohmic losses inside the sphere and normalized in the same way. Conservation of energy leads to *R*+*T*+*A*=1, condition that allows internal check of consistency. These results were also checked by performing the same calculation in CST Microwave Studio, showing excellent agreement.

Simulations of silicon disk arrays over substrate (with interface in *z*=0 and refractive index *n*=1.45) were carried out using the same approach as described for spheres. The main difference is that, in this case, Fresnel equations were used to explicitly write the excitation fields in the upper (*z*>0) and lower (*z*<0) half spaces. While transmission and absorption are computed in exactly the same way, for reflection calculations one needs to consider the Poynting vector of the scattered fields plus the reflected fields from the substrate. In these simulations the refractive index of amorphous silicon^40^ was used to approximate the deposited amorphous silicon in the experiment.

### Multipole decomposition

Multipole decomposition technique was employed to analyse the different modes being excited in the particles. For particles in an array embedded in air, multipoles can be computed through the polarization currents induced within them:


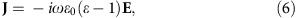


where *ɛ* is the permittivity of the particle and **E**=**E**(**r**) is the electric field inside it.

This approach fully takes into account mutual interactions in the lattice^41^ as well as the possible presence of a substrate. In particular, a Cartesian basis with origin in the centre of the particles was used in the present work. An accurate description of the radiative properties in this basis involves the introduction of the family of toroidal moments^42^,^43^,^44^ and the mean-square radii corrections. The explicit expressions of the multipoles as well as the associated partial scattering cross section can be found in Supplementary Note 9.

### Nanodisk array fabrication

Thin films of amorphous silicon of desired thickness were deposited on fused silica substrates via electron-beam evaporation (Angstrom Engineering Evovac). The samples were then patterned by single-step electron-beam lithography: by spin-coating HSQ resist (Dow Corning, XR-1541-006) and a charge-dissipation layer (Espacer 300AX01), e-beam patterning of the resist (Elionix ELS-7000), and subsequent etching via reactive-ion-etching in inductively coupled plasma system (Oxford Plasmalab 100). The remaining HSQ resist (∼50 nm after etching) on the top of the nanodisks was not removed since its optical properties after e-beam exposure are close to that of silicon dioxide. To reduce losses the fabricated sample was annealed in vacuum at 600 °C for 40 min by using Rapid Thermal Process system (Model: JetFirst200).

### Optical measurements

Transmission and reflection measurements of the nanodisk arrays at normal incidence were conducted using an inverted microscopy set-up (Nikon Ti-U). For transmission measurements, light from a broadband halogen lamp was normally incident onto the sample from the substrate side before being collected by a × 5 objective (Nikon, NA 0.15) and routed to a spectrometer (Andor SR-303i) with a 400 × 1,600 pixel EMCCD detector (Andor Newton), as described in detail elsewhere^15^. Transmitted light through the array was normalized to the transmitted power through the substrate only, after accounting for photodetector noise effects (dark current subtraction). For reflection measurements, light from the broadband halogen lamp was incident into the nanodisk array directly passing through the × 5 objective. The reflected light was then collected by the same objective and routed into the spectrometer. Reflected light from the array was normalized to the incident power, which is characterized by the reflection of a silver mirror with known spectral response.

Angular transmission and reflection measurements were performed using a home-built free-space microscopy set-up. Light originating from a supercontinuum source (SuperK Power, NKT Photonics) was transmitted through a variable band-pass filter for wavelength selection (SuperK Varia, NKT Photonics) and then through a broadband polarizing beam-splitter cube (Thorlabs, PBS252). The linear polarized light passed then through a quarter wave plate (Thorlabs, WPQ10M-808) to obtain circularly polarized light, which was sent to a rotating linear polarizer (Thorlabs, LPNIRE100-B) to obtain linearly polarized light of selected direction. A biconvex lens with 75 mm focusing distance (Thorlabs) was used to focus the light onto the sample surface with silicon nanoparticle arrays. The sample was mounted on a rotation stage for adjusting the angle of incidence. The beam spot size at the sample at normal incidence had a diameter of ∼50 μm being smaller than the size of the fabricated arrays (100 × 100 μm). A white-light lamp source was also coupled into the beam path through the same broadband polarizing beam-splitter cube for sample imaging. Both the incident beam power and the transmitted/reflected beam power were measured by a pixel photodetector attached to a digital handheld laser power/energy metre console (Thorlabs, PM100D). A scheme of the experimental set-up is included in Supplementary Fig. 11 and described in Supplementary Note 10.

## Additional information

**How to cite this article:** Paniagua-Domínguez, R. *et al.* Generalized Brewster effect in dielectric metasurfaces. *Nat. Commun.* 7:10362 doi: 10.1038/ncomms10362 (2016).

## Supplementary Material

Supplementary Information Supplementary Figures 1 - 11, Supplementary Table 1, Supplementary Notes 1 - 10 and Supplementary References.
