# Supplementary material for: Generalized Brewster effect in dielectric metasurfaces
Source: Nat Commun. 2016 Jan 19;7:10362. doi: 10.1038/ncomms10362 (PMC4735648; doi:10.1038/ncomms10362)
Supplement: Supplementary Information — Supplementary Figures 1 - 11, Supplementary Table 1, Supplementary Notes 1 - 10 and Supplementary References. [file ncomms10362-s1.pdf]

Supplementary Figures:

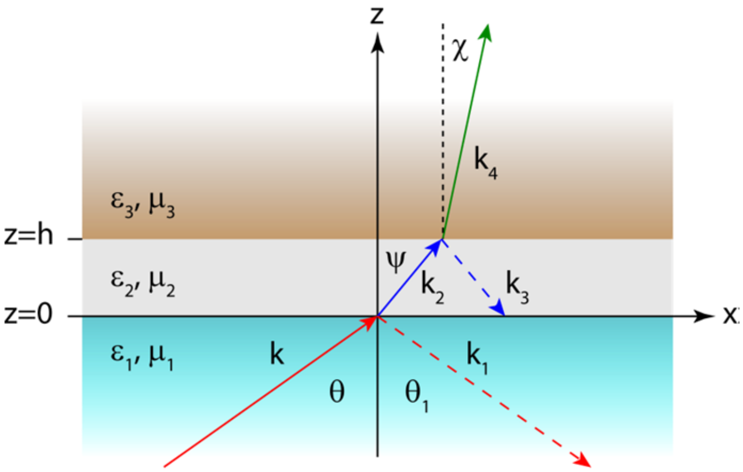

Supplementary Figure 1. Schematics of light transmission and reflection from a slab confined between two infinite media.

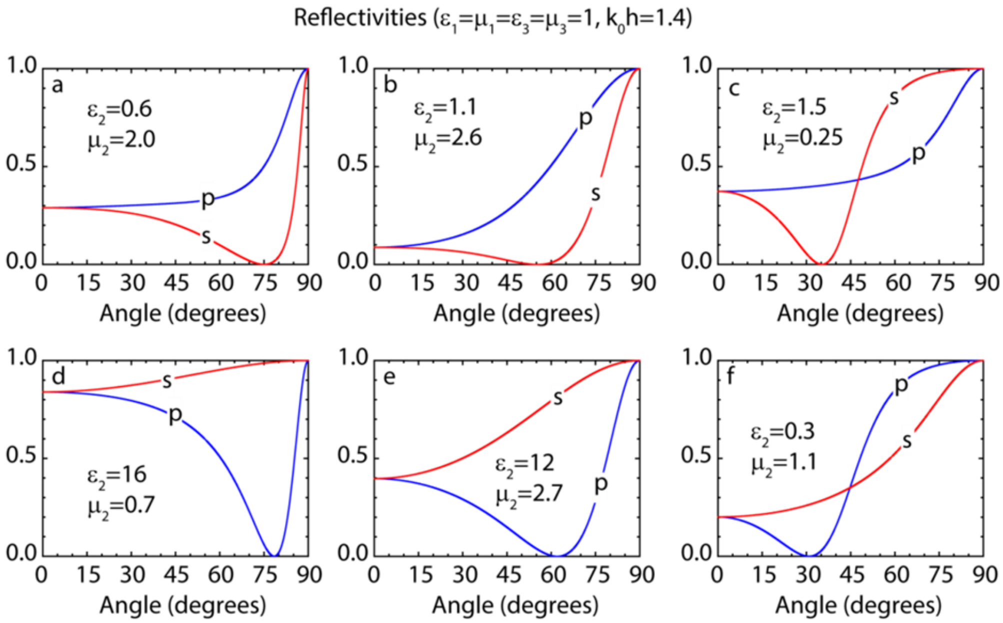

Supplementary Figure 2. Reflectivity of a magneto-electric slab in vacuum: $\boldsymbol{\varepsilon_1 = \mu_1 = \varepsilon_3 = \mu_3 = 1}$ and $\boldsymbol{k_0 h = 1.4}$ (corresponding to $\boldsymbol{h = 150}$ nm at wavelength $\boldsymbol{\lambda \sim 670}$ nm) vs angle of incidence for different $\boldsymbol{\varepsilon_2}$ and $\boldsymbol{\mu_2}$. a-c Brewster's angle for *s*-polarization; **d-f** Brewster's angle for *p*-polarization. Arbitrary values of Brewster's angle can be obtained between 0° and 90° without having total internal reflection at larger angles.

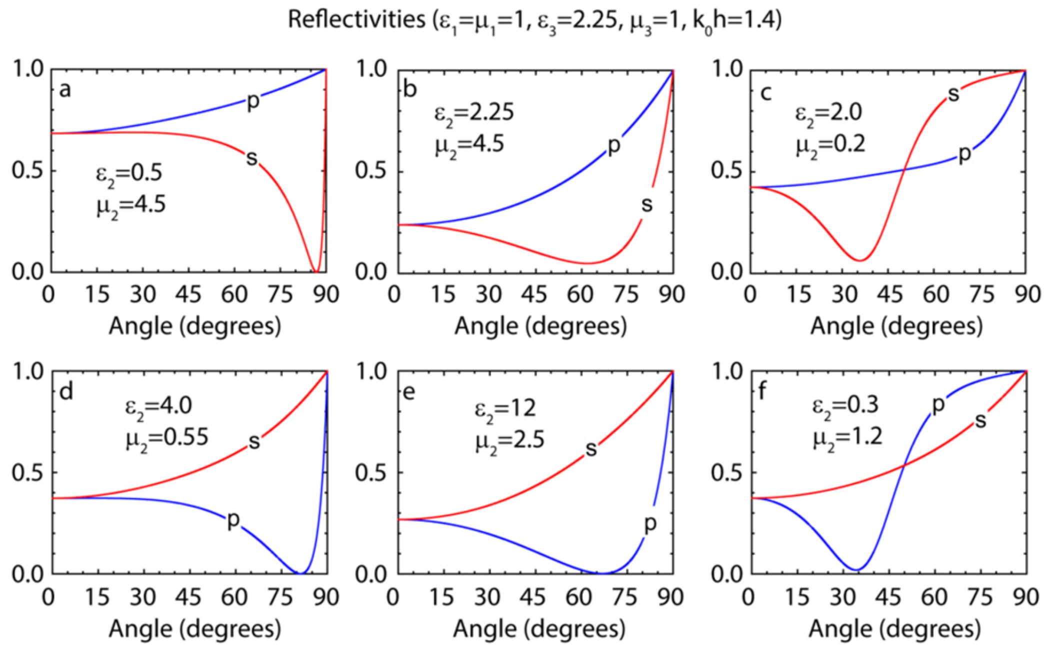

Supplementary Figure 3. Reflectivity of a magneto-electric slab on top of glass: $\boldsymbol{\varepsilon_1 = \mu_1 = 1}$, $\boldsymbol{\varepsilon_3 = 2.25}$, $\boldsymbol{\mu_3 = 1}$ and $\boldsymbol{k_0 h = 1.4}$ (corresponding to $\boldsymbol{h = 150}$ nm at wavelength $\boldsymbol{\lambda \sim 670}$ nm) vs angle of incidence for different $\boldsymbol{\varepsilon_2}$ and $\boldsymbol{\mu_2}$. a-c Brewster's angle for *s*-polarization; **d-f** Brewster's angle for *p*-polarization. Arbitrary values of Brewster's angle can be obtained (0° - 90°) without having total internal reflection.

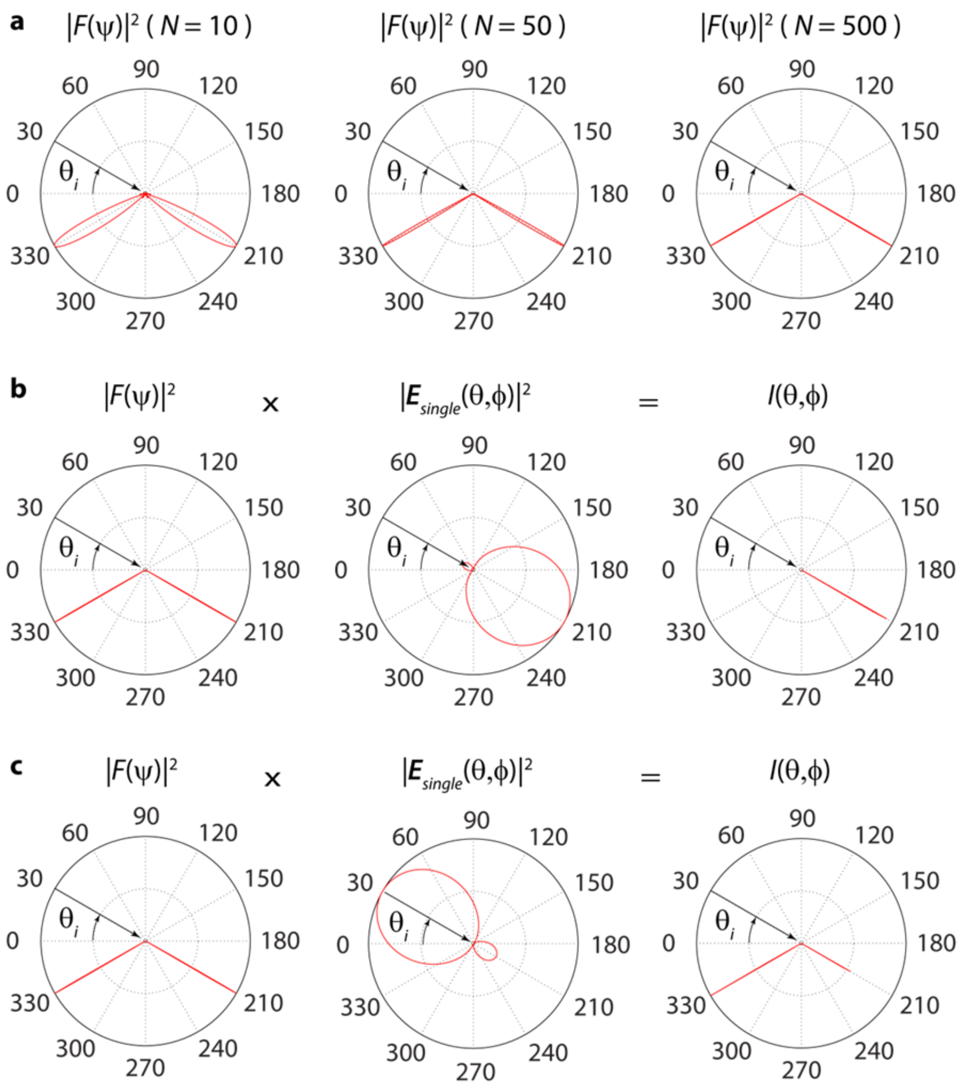

Supplementary Figure 4. Scattering properties of a phased array of identical electric and magnetic dipoles. a, The form factor $|F(\psi)|^2$ of the array as a function of the number of particles considered. The form factor quickly converges towards zero everywhere except at the reflection and transmission direction, for which $|F(\psi)|^2 = 1$. The direction of incidence is indicated by a black arrow together with the incidence angle $\theta_i = \pi/6$. The wavelength is $\lambda = 730$ nm and the pitch $d = 300$ nm. **b**, The form factor $|F(\psi)|^2$ (left), the radiation pattern of each single element in the array $\mathbf{E}_{\mathrm{single}}(\theta, \phi)$ (center) and the total radiated intensity from the array $I(\theta, \phi)$ (right) for an array of electric and magnetic dipoles with $\mathbf{m} = (-m \cdot c \cos\theta_{\mathrm{i}},\ 0,\ m \cdot c \sin\theta_{\mathrm{i}})$ and $\mathbf{p} = (0, -p, 0)$, fulfilling the condition $p = m/2$. Parameters λ, θ_{i} and d are the same as in **a**, $N = 500$. This configuration suppresses reflection in *s*-polarization. **c**, The same as in b but with $p = m/3$ for which reflection is not suppressed.

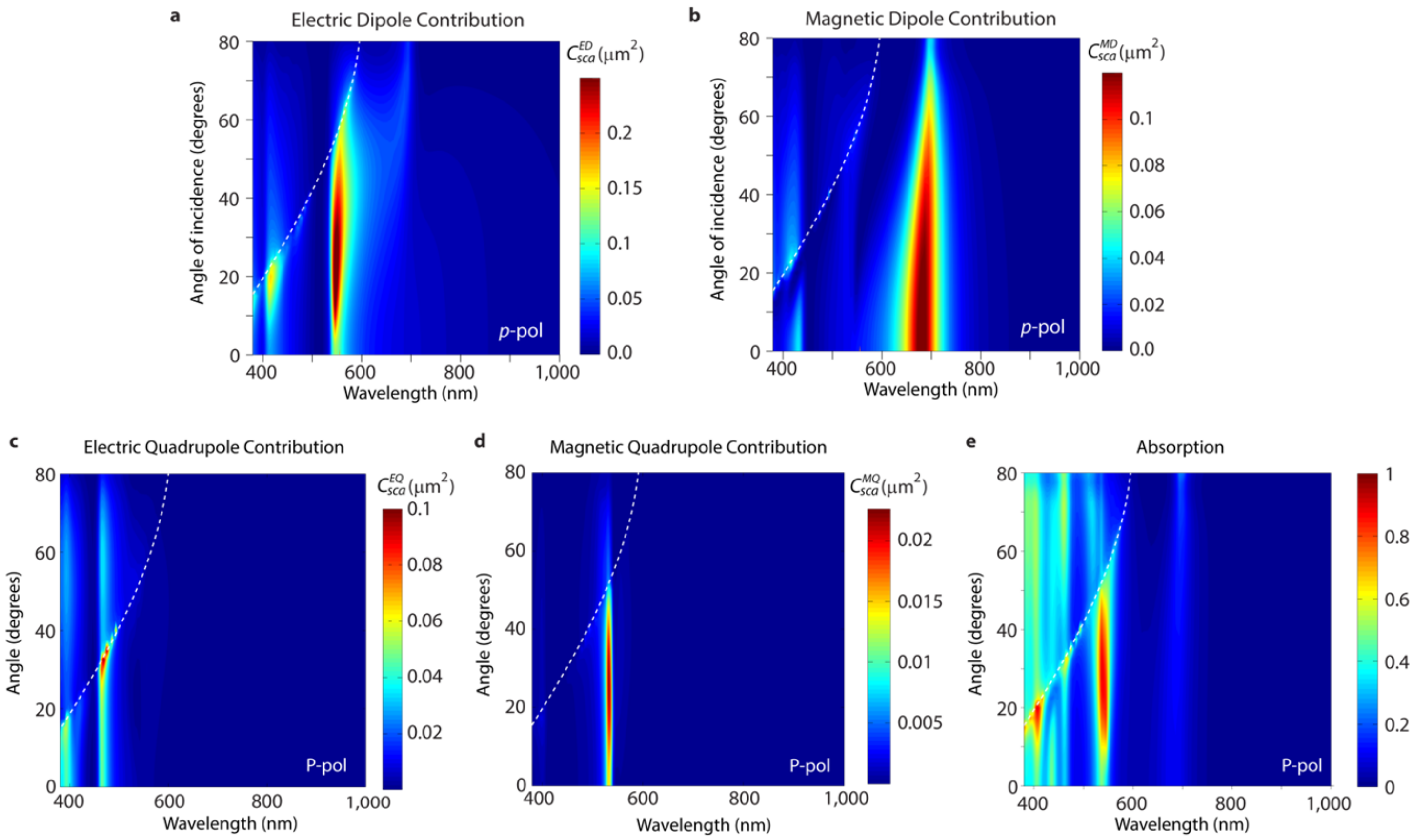

Supplementary Figure 5. Multipolar contributions and absorption in a square lattice of silicon spheres versus wavelength and angle of incidence under *p*-polarized incidence. a, and **b,** respectively, electric and magnetic dipolar contributions to the scattering from each particle in the array as retrieved by multipole decomposition. Dashed white lines show the spectral position of the first diffraction order. **c** and **d**, respectively, electric and magnetic quadrupolar contributions to the scattering from a particle in the array as retrieved by multipole decomposition. **e**, Absorption computed through volume integration of the Ohmic losses.

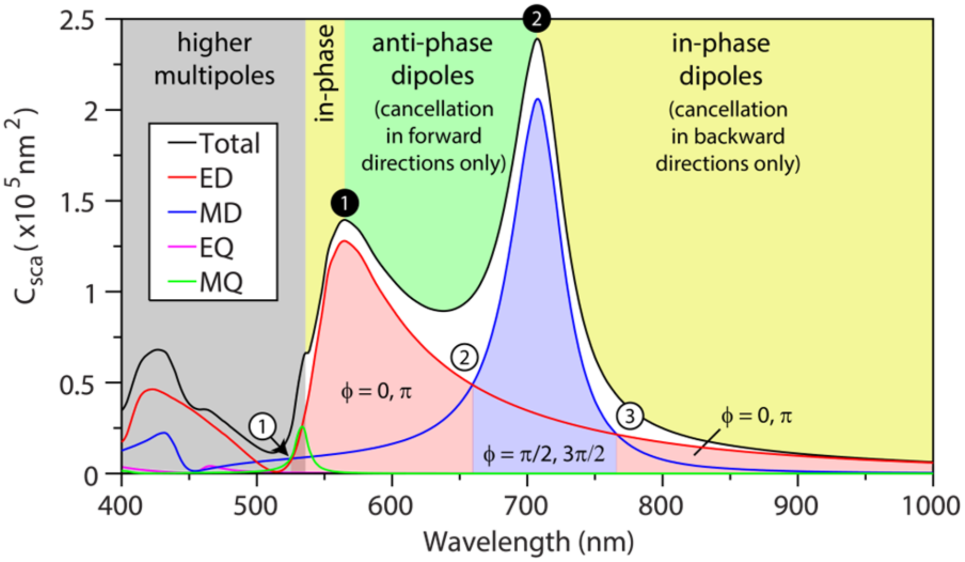

Supplementary Figure 6. Optical properties of a silicon sphere with diameter $D = 180$ nm in air under plane wave illumination, and regions and points of interest. Total scattering cross section (black curve) and contributions from the electric dipole (red curve), magnetic dipole (blue curve), electric quadrupole (magenta curve) and magnetic quadrupole (green curve).

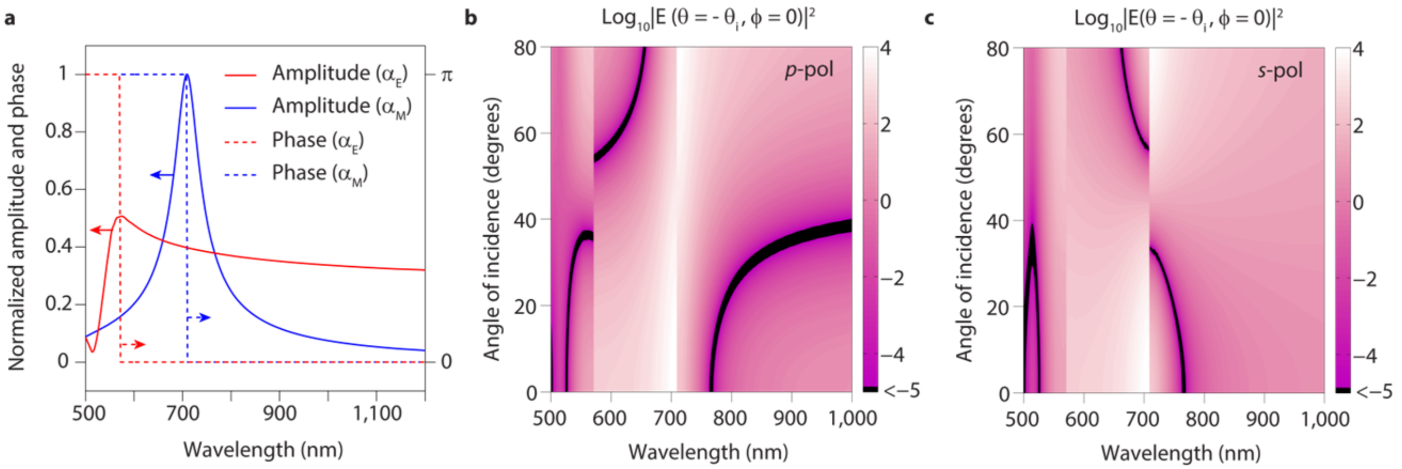

Supplementary Figure 7. Radiated intensity in the reflection direction by a pair of electric and magnetic dipoles under *p*-polarized (red) and *s*-polarized (blue) light. a, Amplitudes and phases of the polarizabilities of the dipoles. **b,** Radiated intensity in the plane of incidence ($\phi = 0$) and reflection direction ($\theta = -\theta_{\mathrm{i}}$) by the pair of dipoles under *p*-polarization. **c,** The same as in **b** but for *s*-polarization.

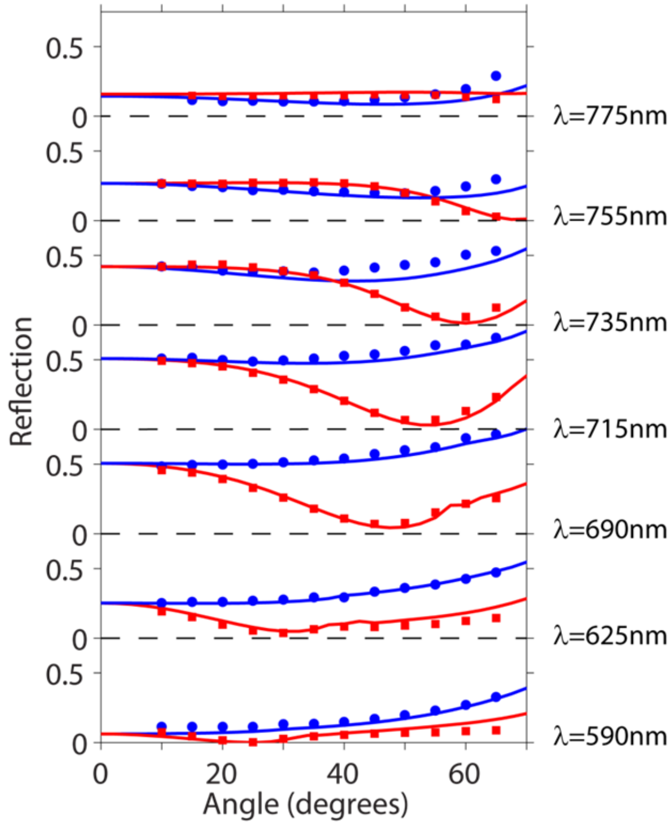

Supplementary Figure 8. Reflection vs angle of incidence under *p*-polarized (red) and *s*-polarized (blue) light in a square lattice of a-silicon nanodisks over a fused silica substrate. The plot shows the comparison between measured values (symbols) and simulations (lines) obtained fitting the imaginary part of the index (n''). Real part (n') was taken from Ref.4.

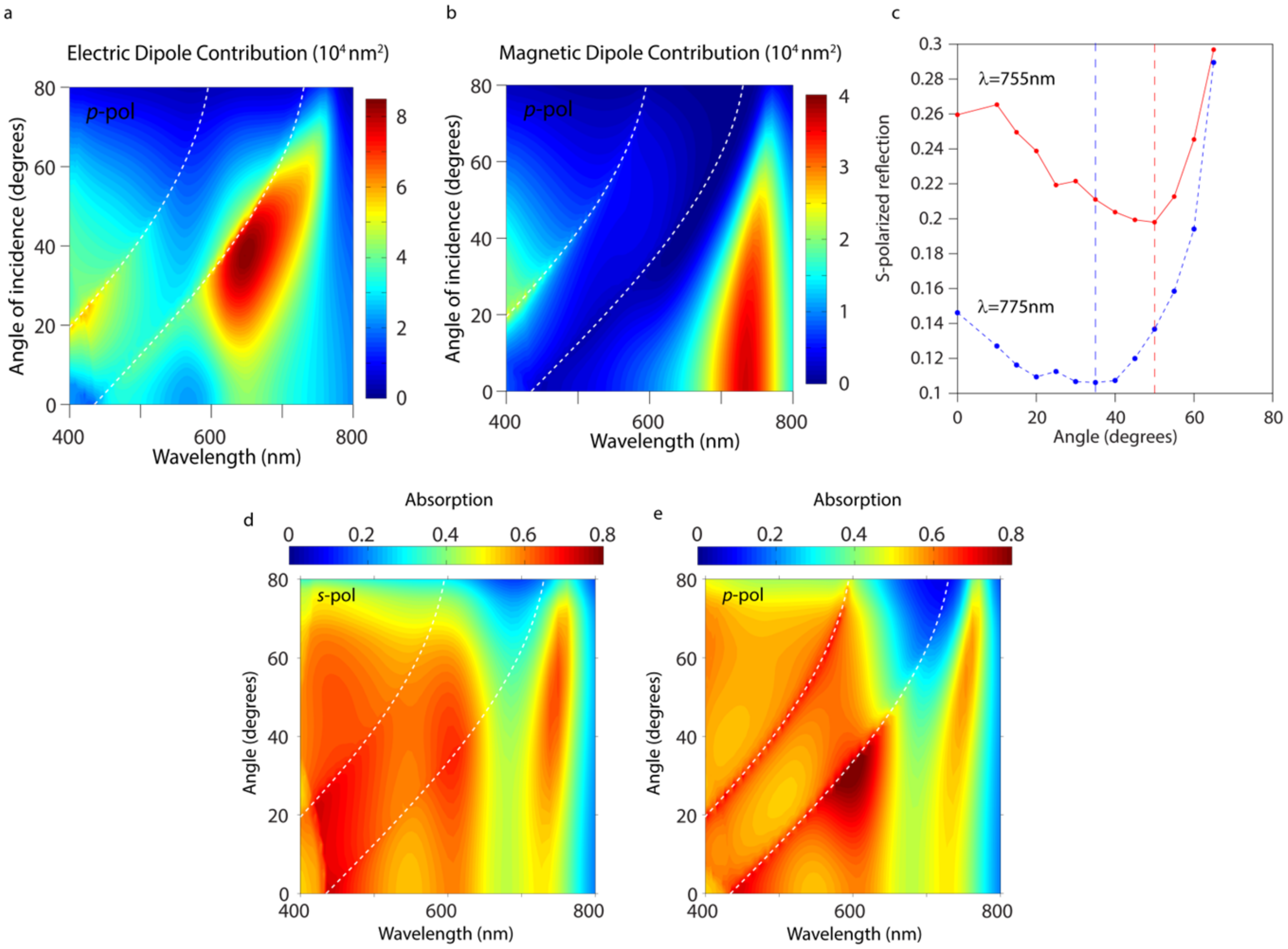

Supplementary Figure 9. Multipolar contributions from a single disk in the array under *p*-polarized incidence, detail of reflection under *s*-polarized light and absorption in both polarizations in a square lattice of silicon nanodisks over a fused silica substrate versus angle of incidence and wavelength. a-b, Simulated electric (a) and magnetic (b) dipole contributions to the total scattering cross section of a single disk in the square lattice of silicon nanodisks over a fused silica substrate under p-polarized light. **c,** Details of the measured angular reflection from the array under s-polarized light for particular wavelengths. **d, e,** Simulated absorption computed through volume integration of the ohmic losses inside the particles, under *s*- and *p*-polarized incident light, respectively.

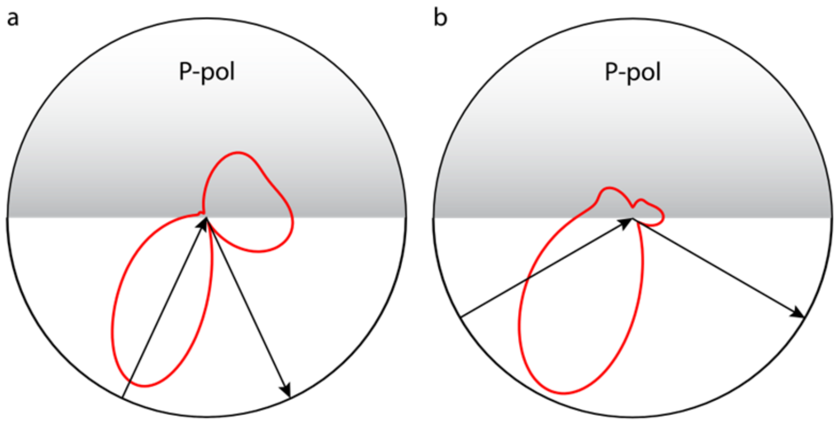

Supplementary Figure 10. Radiation patterns in the plane of incidence from electric and magnetic dipoles excited in each silicon nanodisk in the square array of the nanodisks on silica substrate under *p*-polarized incident light. a, $\boldsymbol{\lambda = 590}$ nm, when reflection vanishes at the incidence angle of 25 degrees. **b**, $\boldsymbol{\lambda = 735}$ nm, when reflection vanishes at the incidence of 60 degrees.

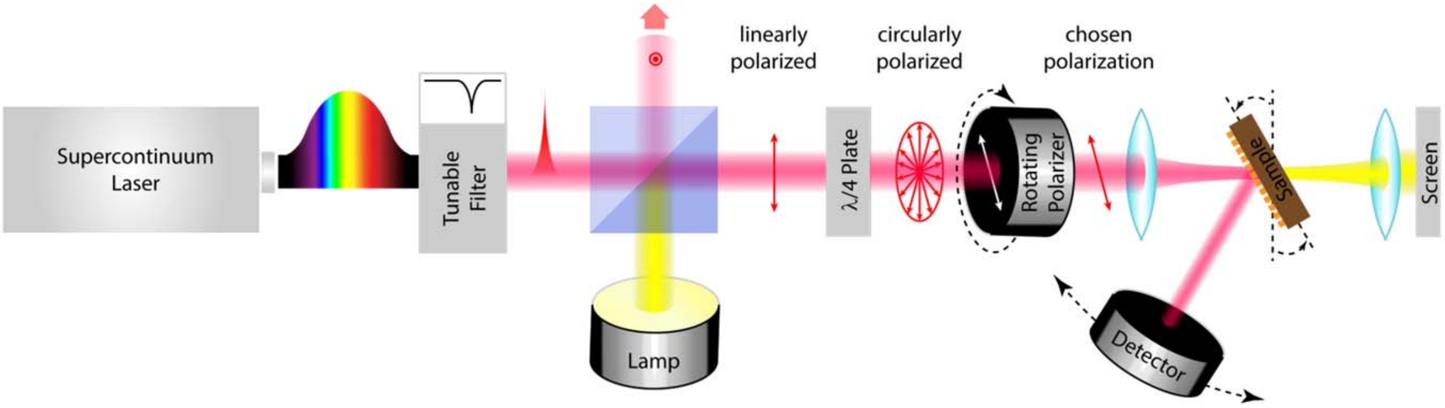

Supplementary Figure 11. Scheme of the home-built experimental setup used for measuring angular transmission/reflection from the fabricated nanodisks arrays.

Supplementary Tables:

**Wavelength (nm)**	n'' **(fitting)**	n'' **(Ref.4)**
**590**	**0.583**	**0.583**
**625**	**0.300**	**0.445**
**690**	**0.140**	**0.269**
**715**	**0.135**	**0.224**
**735**	**0.130**	**0.191**
**735**	**0.130**	**0.164**
**755**	**0.130**	**0.136**

Supplementary Table 1. Values of imaginary part of refractive index used in simulations in Supplementary Fig.8 and those from Ref.4.

Supplemetary Note 1: Generalized Brewster's effect for a magneto-electric slab.

The present section aims to present the not-so-well-known rich phenomenology associated with reflection of plane waves at an interface between an ordinary medium and one having simultaneous electric and magnetic responses. Instead of analyzing a single interface, let us focus on the case of a slab, located either in air or standing over a semi-infinite glass, since these cases arguably model more accurately the system studied in the main manuscript.

Consider the general case represented in Supplementary Fig.1. The thickness of the film with optical properties given by ε_2 and μ_2 is h, while media 1 and media 3 are semi-infinite. As usual, two different polarizations (*p*- and *s*-) should be considered.

Similar to the case of ordinary dielectrics, Fresnel's formulas follow from the boundary conditions of electric and magnetic fields at interfaces $z=0$ and $z=h$ (continuity of the tangential field components). This yields four equations for the *y*-components of the magnetic or electric fields (for *p* or *s*-polarization, respectively). Solving these equations the formulas for the amplitudes of the reflection coefficients are obtained.

a) ***p-polarization***. The reflection coefficient is given by $r_{\mathrm{p}} = H_{1y}/H_{0y}$, where H_{0y} and H_{1y} are the magnetic field amplitude of the forward (incident) and backward (reflected) propagating waves in media 1. The reflectivity is given by $\mathfrak{R}_{\mathrm{p}} = |r_{\mathrm{p}}|^2$. This formula can be presented in standard form[1]:

$$r_{\mathrm{p}} = \frac{r_{12}^{(p)} e^{-2i\psi} + r_{23}^{(p)}}{e^{-2i\psi} + r_{12}^{(p)} r_{23}^{(p)}}, \tag{1}$$

where the amplitude reflection coefficient r_{12} (between media 1 and media 2) is given by:

$$r_{12}^{(p)} = \frac{\varepsilon_2\sqrt{\mu_1}\cos\theta - \sqrt{\varepsilon_1}\sqrt{\varepsilon_2\mu_2 - \varepsilon_1\mu_1\sin^2\theta}}{\varepsilon_2\sqrt{\mu_1}\cos\theta + \sqrt{\varepsilon_1}\sqrt{\varepsilon_2\mu_2 - \varepsilon_1\mu_1\sin^2\theta}} \tag{2}$$

and the amplitude reflection coefficient r_{23} (between media 2 and media 3) is given by:

$$r_{23}^{(p)} = \frac{\varepsilon_3\sqrt{\varepsilon_2\mu_2 - \varepsilon_1\mu_1\sin^2\theta} - \varepsilon_2\sqrt{\varepsilon_3\mu_3 - \varepsilon_1\mu_1\sin^2\theta}}{\varepsilon_3\sqrt{\varepsilon_2\mu_2 - \varepsilon_1\mu_1\sin^2\theta} + \varepsilon_2\sqrt{\varepsilon_3\mu_3 - \varepsilon_1\mu_1\sin^2\theta}} \tag{3}$$

and $\psi = \psi(\theta)$ represents the change in the wave phase over the thickness h of the layer ($k_0 = \omega/c$):

$$\psi = k_0 h\sqrt{\varepsilon_2\mu_2 - \varepsilon_1\mu_1 \sin^2\theta} \tag{4}$$

b) *s-polarization*. In a totally analogous way, the reflection coefficient is given by $r_s = E_{1y}/E_{0y}$, and the reflectivity is $\Re_s = |r_s|^2$. In this case:

$$r_s = -\frac{r_{12}^{(s)}e^{-2i\psi} + r_{23}^{(s)}}{e^{-2i\psi} + r_{12}^{(s)}r_{23}^{(s)}}, \tag{5}$$

where:

$$r_{12}^{(s)} = \frac{\mu_2\sqrt{\varepsilon_1}\cos\theta - \sqrt{\mu_1}\sqrt{\varepsilon_2\mu_2 - \varepsilon_1\mu_1\sin^2\theta}}{\mu_2\sqrt{\varepsilon_1}\cos\theta + \sqrt{\mu_1}\sqrt{\varepsilon_2\mu_2 - \varepsilon_1\mu_1\sin^2\theta}}, \tag{6}$$

$$r_{23}^{(s)} = \frac{\mu_3\sqrt{\varepsilon_2\mu_2 - \varepsilon_1\mu_1\sin^2\theta} - \mu_2\sqrt{\varepsilon_3\mu_3 - \varepsilon_1\mu_1\sin^2\theta}}{\mu_3\sqrt{\varepsilon_2\mu_2 - \varepsilon_1\mu_1\sin^2\theta} + \mu_2\sqrt{\varepsilon_3\mu_3 - \varepsilon_1\mu_1\sin^2\theta}}, \tag{7}$$

Note that amplitude coefficients of reflection r_p and r_s in the limiting case $\theta = 0$ differ in sign, as **E** represents a polar, and **H** an axial vector[2].

From this analysis it is readily seen that with appropriate variations of ε and μ it is possible to obtain arbitrary values for the Brewster angle, corresponding to the vanishing value of reflectivity, both for *p*-polarized light and for *s*-polarized light.

We illustrate now the phenomenology associated with the generalized Brewster's effect by considering a slab with ε and μ standing in air. Results, shown in Supplementary Fig.2, are selected to illustrate the main characteristics of the generalized Brewster, namely, the possibility to obtain Brewster angle for s-polarized light (Supplementary Fig.1a-c) and the possibility to obtain Brewster for angles below 45 degrees without having total internal reflection (TIR) for larger angles (Supplementary Figs. 2c and f). Note that this phenomenology is analogous to that observed in the case of sub-diffractive silicon nanosphere array embedded in air, as presented in section 1 of the main text.

For the sake of completeness we illustrate, in Supplementary Fig.3 the case of a magneto-electric slab on top of a glass semi-infinite medium (incidence from the side of air). As readily seen, this configuration retains all major characteristics, and serves to illustrate the phenomenology found in the case of silicon nanoparticle array over glass substrate presented in section 2 of the main text.

Supplementary Note 2: Electric far-field radiated by a pair of electric and magnetic dipoles.

Consider a pair of electric and magnetic dipoles. The electric far-field radiated in the direction given by the unit vector $\hat{\boldsymbol{n}}$ can be written as:

$$\mathbf{E}_{\text{ff}} = \mathbf{E}_{\text{ff}}{}^{\text{p}} + \mathbf{E}_{\text{ff}}{}^{\text{m}} = \frac{k_0^2}{4\pi\epsilon_0}\left[\hat{\mathbf{n}} \times (\mathbf{p} \times \hat{\mathbf{n}}) + \frac{1}{c}\mathbf{m} \times \hat{\mathbf{n}}\right] \tag{8}$$

with $k_0 = 2\pi/\lambda$ the wavenumber and ϵ_0 and c the permittivity and speed of light in vacuum, respectively.

Consider the situations depicted in Fig.2a of the main text. The induced dipoles, oscillating parallel to the driving incident fields can be written as $\boldsymbol{p} = (-p\,cos\,\theta_i\,,\ 0,\ p\,sin\,\theta_i)$ and $\boldsymbol{m} = (0,\ m \cdot c,\ 0)$, with p and m the complex amplitudes of the induced dipoles and θ_i the angle of incidence. In this situation, the radiated (scattered) field in a direction of observation given by the polar angle θ in the *xz*-plane (highlighted in the figure) is purely polar and reads:

$$\mathbf{E}_{\text{ff}} \propto [m - p\cos(\theta - \theta_{\text{i}})]\hat{\boldsymbol{\theta}}, \tag{9}$$

with $\hat{\boldsymbol{\theta}}$ the unitary polar vector. It is clear that, in this situation, the electric field is suppressed if:

$$\cos(\theta - \theta_{\text{i}}) = m/p = (|m|/|p|)e^{i\delta} \tag{10}$$

with $\boldsymbol{\delta}$ being the phase difference between the two dipoles. Whenever the phase difference between dipoles is a multiple of the field exactly vanishes. It is clear that, when an infinite array of spheres is considered in the xy-plane, this situation represents the *p*-polarization incidence case, and the plane of incidence coincides with the xz-plane.

Analogously, when the case depicted in Fig.2b holds, the induced electric and magnetic dipoles can be described by $\mathbf{m} = (-\boldsymbol{m} \cdot \boldsymbol{c}\,\mathbf{cos}\,\boldsymbol{\theta}_{\mathbf{i}}\,, \mathbf{0}, \boldsymbol{m} \cdot \boldsymbol{c}\,\mathbf{sin}\,\boldsymbol{\theta}_{\mathbf{i}})$ and $\mathbf{p} = (\mathbf{0}, -\boldsymbol{p}, \mathbf{0})$. In this situation, the radiated (scattered) field in the plane containing the magnetic dipole (highlighted in Fig.2b) is purely azimuthal and reads:

$$\mathbf{E}_{\text{ff}} \propto [p - m\cos(\theta - \theta_{\text{i}})]\hat{\boldsymbol{\phi}} \tag{11}$$

with $\hat{\boldsymbol{\phi}}$ the unitary azimuthal vector. It vanishes if:

$$\cos(\theta - \theta_{\text{i}}) = p/m = (|p|/|m|)e^{-i\delta}, \tag{12}$$

and will represent the *s*-polarization case for infinite arrays.

Supplementary Note 3: Phased arrays of point scatters.

It is known from the phased array antennas theory that the total intensity from an array of identical emitters can be expressed as:

$$I(\theta,\phi) = |F(\psi)|^2 \left|\mathbf{E}_{\text{single}}(\theta,\phi)\right|^2 \tag{13}$$

where $\boldsymbol{F(\psi)}$ is the so called form factor of the array, which describes the phase retardation from different elements in the lattice and $\mathbf{E_{single}}(\boldsymbol{\theta},\boldsymbol{\phi})$ is the far-field of each identical constituent. An analogous formula holds to describe the scattering properties of an array of identical point-like scatters. As in the case of phased array antennas, $\boldsymbol{F(\psi)}$ carries information about the geometry of the array and does not depend on the particular scatters considered. It reads:

$$F(\psi) = \frac{\sin\left(\frac{N\psi}{2}\right)}{N\sin\left(\frac{\psi}{2}\right)} \tag{14}$$

in which $\boldsymbol{\psi = kd\sin\theta\cos\phi + \xi}$, with $\boldsymbol{k = 2\pi/\lambda}$ being the wavenumber, $\boldsymbol{d}$ the lattice period and $\boldsymbol{\xi = kd\sin\theta_i}$ the phase difference due to oblique incidence at an angle $\boldsymbol{\theta_i}$ (we consider the plane of incidence as $\boldsymbol{\phi = 0}$). Here *N* is the number of particles in the array. In the limit $\boldsymbol{N} \to \infty$one has:

$$\lim_{N\to\infty} F(\psi) = \begin{cases} 1, & \psi \to\ 0 \\ 0, & \psi \nrightarrow\ 0 \end{cases} \tag{15}$$

Fixing the scattering plane to $\phi = 0$, $F(\psi)$ is non-zero only when:

$$\text{Sin}\theta + \sin\theta_\text{i} = 2\sin\left(\frac{\theta+\theta_\text{i}}{2}\right)\cos\left(\frac{\theta-\theta_\text{i}}{2}\right) = 0. \tag{16}$$

This implies that, if no higher diffracted order are present, $F(\psi)$ is non-zero when $\theta = -\theta_\text{i} \equiv \theta_\text{r}$ or $\theta = \pi + \theta_\text{i} \equiv \theta_\text{t}$. In Supplementary Fig.4a, $|F(\psi)|^2$ is plotted for $\theta_\text{i} = \pi/6$, $\lambda = 730$ nm and $d = 300$ nm for several increasing number of particles N. As seen, it quickly converges to the limit above, vanishing everywhere except in the reflection and transmission directions.

Let us now assume that each single element in the array is a pair of electric ($\mathbf{p}$) and magnetic ($\mathbf{m}$) dipoles. The radiated far-field $\mathbf{E}_{\text{single}}(\theta,\phi)$ will be given by equation (8), see Supplementary Note 2 above. Consider the two main situations presented there. In the first the electric dipole is contained in the plane of incidence ($\phi = 0$) with $\mathbf{p} = (-p\cos\theta_{\text{i}},\ 0,\ p\sin\theta_{\text{i}})$, and $\mathbf{m} = (0,\ m\cdot c,\ 0)$. In this case, the radiated field in this plane is given by (9). Clearly, this situation will represent the case of *p*-polarized incidence. In the second case, the magnetic dipole is contained in the incidence plane and reads $\mathbf{m} = (-m\cdot c\cos\theta_{\text{i}},\ 0,\ m\cdot c\sin\theta_{\text{i}})$ while $\mathbf{p} = (0, -p, 0)$. In this situation the radiated field in the plane of incidence is given by (11) and will represent the case of s-polarized incidence.

From (10) and (12) one can compute the relative values of p and m for which the field at $\theta = \theta_{\text{r}} = -\theta_{\text{i}}$ will be zero. In this case, no intensity at all will be radiated in the reflection direction (as follows from equation (13)), leading to perfect transmission, i.e., to Brewster's effect. Let us consider, e.g. the case of $\theta_i = \pi/6$ and *s*-polarization. From equation (12S) it follows that the field vanishes at $\theta = -\theta_{\text{i}} = -\pi/6$ for $p = m/2$. Note that this relation immediately implies $C_{\text{sca}}^{\text{ED}}/C_{\text{sca}}^{\text{MD}} = 1/4$ (see equations (4) and (5) of the main text), which precisely corresponds to the case depicted in the bottom panel of Fig.2c in the main text. In Supplementary Fig.4b we plot $|F(\psi)|^2$ (left), $\left|\mathbf{E}_{\text{single}}(\theta,\phi)\right|^2$ (center) and $I(\theta,\phi)$ (right) for this case (with $\theta_i = \pi/6$, $\lambda = 730$ nm, $d = 300$ nm and $N = 500$). It is clear from the calculation that, due to the modulation of the form factor $|F(\psi)|^2$, radiation in any other direction rather than those of transmission and reflection is totally inhibited due to interference from different lattice sites, even if the single particles radiate in those directions. It is also immediately seen that the suppression of radiation in the reflection direction from each single element implies the suppression of radiation from the whole array. Finally, to stress the origin of the effect in the inhibition of radiation from single elements, we plot in Supplementary Fig.4c the case $p = m/3$. This ratio does not lead to zero radiation in the reflection direction and, thus, no Brewster is obtained.

Supplementary Note 4: Absorption and higher order multipoles in arrays of spheres at *p*-polarized incidence.

It is our intention here to complete the picture given in Section 1 of the main manuscript, regarding the analysis of the resonances excited in the array of silicon (Si) spheres with diameter $D = 180$ nm and pitch $P = 300$ nm for different wavelengths and angles of incidence for *p*-polarized light. As mentioned in the main text, the electric and magnetic dipole contributions are the dominant ones in the range of wavelengths and angles of incidence studied. Those are shown in the whole simulated range in Supplementary Fig.5a and b, respectively. Also, the electric quadrupole partial scattering cross section, as computed through the multipole decomposition, is shown in Supplementary Fig.5b, while the corresponding plot for the magnetic quadrupole is shown in Supplementary Fig.5c. As readily seen, both resonances appear for wavelengths much shorter than those for which the generalized Brewster effect is observed. Supplementary Figure 5d also shows the absorption in the array, computed through volume integration of the Ohmic losses inside the spheres. These results serve first as a demonstration of the energy conservation in our simulations and also to track all resonances excited in the system, showing excellent correspondence with those computed through the multipole decomposition technique (the magnetic one, however, is fainter due to the lower dissipation of silicon at that wavelength).

Supplementary Note 5: Interpretation of the observed generalized Brewster effect based on the scattering characteristics of a single sphere.

Let us consider more carefully the scattering characteristics of a single silicon sphere, Supplementary Fig.6 (the figure is similar to Fig.2b in the main manuscript, but some points of interest are added). Although the real picture is more complicated due to the inter-particle interactions inside the arrays, a simplified model of a single sphere may reveal major features of the reflection maps in Figs.3b and 4b from the main manuscript. In this spirit let us assume here that particles in the lattice behave similar to individual scatters. In this case, as already mentioned, cancellation of radiation in the plane containing the incident electric field (corresponding to

the case of *p*-polarized incidence) is only possible when the induced electric dipole dominates, i.e., in the red shaded regions in Supplementary Fig.6. Cancellation in the perpendicular plane (corresponding to the case of *s*-polarized incidence) is only possible under the blue shaded regions (dominance of magnetic dipole). Since the scattering angle and the reflection angle are related as $\theta = -\theta_i$, it is clear that for $\theta_i < 45$ the dipoles must be in phase (yellow shaded regions) while for $\theta_i > 45$ they must be in anti-phase (green shaded region).

Let us start by analyzing the *p*-polarized case. Thus, to achieve the zero reflection effect we must restrict ourselves to the red shaded regions where electric dipole dominates. At normal incidence the first Kerker's condition, indicated by ① and ③ in Supplementary Fig.5, leads to zero reflection. Let us first analyze the spectral region around ①. For increasing angles of incidence, equation (4) implies that $C_{\mathrm{sca}}^{\mathrm{MD}}/C_{\mathrm{sca}}^{\mathrm{ED}}$ should correspondingly decrease. This is achieved at longer wavelengths with respect to ①, which manifests as the slight redshift in the zero of reflection in Fig.3b (region indicated by the white dashed curve 1) for angles below 45 degrees. Above 45 degrees, the dipoles have to be in opposite phases to cancel radiation in the reflection direction, thus crossing ❶ in Supplementary Fig.6 and entering in the green region, as it is observed in the zero of reflection in Fig.3b (region indicated by the white dashed curve 3). In order to satisfy equation (4) now the rate $C_{\mathrm{sca}}^{\mathrm{MD}}/C_{\mathrm{sca}}^{\mathrm{ED}}$ should increase instead, which is again possible at longer wavelengths. In this region, however, the range of wavelengths is wider going up to ② (above ② MD contribution starts to dominate), leading to a more pronounced redshift in Fig.3b. Thus, the sequence ① → ❶ → ② always implies a redshift to fulfill equation (4), as observed in Fig.3b. Interestingly, if now ③ is chosen as the starting point, fulfilling equation (4) again implies longer wavelengths for larger angles of incidence. However, cancellation is only possible below 45 degrees, since there is no region in which the dipoles are in anti-phase, thus explaining the asymptotic behavior of the zero in reflection < 45 degrees observed in Fig.3b (region indicated by the white dashed curve 2).

Having analyzed the *p*-polarized case, the corresponding analysis of *s*-polarization is straightforward. We are now restricted to move within the blue shaded region. Starting again in Kerker's first condition at normal incidence ③, fulfilling equation (5) now implies a blue-shift. Since at 45 degrees the dipoles must

change from in-phase to anti-phase, the complete sequence is now ③ → ❷ → ②, which implies a constant blue-shift in the whole range, as observed in Fig.4b (region indicated by the white dashed curve 2). Since the blue area is narrower, this directly translates in a narrow spectral band for zero reflection, which in the real system gets reinforced by a narrowing of the magnetic resonance due to the lattice interactions.

Let us conclude showing that the main features observed in the reflectivity of the arrays can be obtained in a simple way from equations (8)-(12), which describe the radiation of a pair of electric and magnetic dipoles. For that, let us assume that the electric (α_{E}) and magnetic (α_{M}) polarizabilities of the dipoles are those of a Si sphere according to Mie theory (i.e., $\alpha_{\mathrm{E}} = 6\pi i a_1/k^3$ and $\alpha_{\mathrm{H}} = 6\pi i b_1/k^3$, with a_1 and b_1 the electric and magnetic dipolar scattering coefficients, respectively[3]). One important assumption is made to correctly reproduce the results. The dipoles are assumed to change their phase abruptly around the resonance peak. For single spheres this only holds approximately but it correctly models the effect of interactions in the array. Of course one could fully take into account the effect of the lattice by computing the self-consistent field at each dipole position and computing the effective polarizabilities. However, it is enough to have good results to consider that the effect of the lattice manifests just as a steeper phase change in the polarizabilities of the particles. Thus, we take the amplitudes as given by Mie theory but assume a step function for the phases, as depicted in Supplementary Fig.7a. Then, by simply applying equations (9) and (11), it is possible to compute the intensity radiated in the reflection angle $\theta = -\theta_{\mathrm{i}}$, as a function of the angle of incidence θ_i and wavelength λ for *p*- and *s*-polarization, respectively. Recall that the condition of zero radiated intensity in the reflection direction is sufficient to have zero reflection (see section 3). The computed intensity is shown in Figs.S7b and c for *p*- and *s*-polarizations. It can be seen that the zones showing zero radiated intensity in the reflection direction closely reproduce those of zero reflectivity found by full numerical simulations (Figs.3b and 4b). For *p*-polarization, it correctly predicts the red-shift of the region of zero reflection at long wavelengths (red side of resonances) as it approaches $\theta_i = 45$ degrees. Also, it reproduces the behavior of the zero reflection region at shorter wavelengths (blue side of the resonances). It predicts its continuous red-shift for increasing angles of incidence and the “jump” to the region between resonances for $\theta_{\mathrm{i}} > 45$. Finally, for s-polarization, it correctly predicts the continuous blue-shift of the zero intensity region located in the red side of the resonances.

Supplementary Note 6: Impact of losses on the angular reflection of a square lattice of nanodisks on top of fused silica. Experiment vs simulations.

In the present section we demonstrate that, as mentioned in the main text, the differences observed between experiment and simulations in the angular reflection of a sample of silicon nanodisks on top of silica substrate are almost entirely due to the lower absorption of the fabricated sample compared to values tabulated[4] for amorphous silicon (a-Si).

As seen in Fig.5 in the main text, the differences are more pronounced above 600nm. Below that limit the agreement is fairly good (see the case at 590nm). Above, however, experiment and theory quickly depart, and reflection is higher in the fabricated sample, indicating a quick drop of absorption as compared to the tabulated data used for simulations.

In Supplementary Fig.8 we show the same set of curves as in Fig.5d of the main manuscript but, instead of directly taking the complex refractive index $(\boldsymbol{n'} + \boldsymbol{in''})$ from Ref.4 we take only the real part $(\boldsymbol{n'})$, and allow the imaginary $(\boldsymbol{n''})$ to be smaller. For each wavelength, we choose it in such a way that measured values show good agreement for low angles of incidence (thus fitting the spectrum at normal incidence). It is readily seen that the agreement between experiment and simulations obtained in this way is excellent.

In Supplementary Table 1 we show the set of values of $\boldsymbol{n''}$ used together with those tabulated. At 590 nm we just took the same value as the tabulated. The mentioned quick drop of the absorption is clearly seen and the value at 775 nm approaches that of Ref.4.

Supplementary Note 7: Multipole contributions and absorption in the square lattice of Si nanodisks on top on fused silica substrate.

In order to complete the analysis of the generalized Brewster's effect for the Si nanodisks metasurface with pitch $P = 300$ nm, diameter $D = 170$ nm and height $H = 160$ nm, given in section 2 of the main manuscript, we present here some additional results. In particular, the electric and magnetic dipolar contributions to the

scattering from a single element in the array as a function of wavelength and angle of incidence under irradiation with p-polarized light are shown in Supplementary Fig.9a and b. Also, we plot in Supplementary Fig.9c the reflection of s- polarized light as a function of angle of incidence for the particular cases of $\lambda = 755$ nm and $\lambda = 775$ nm. It is readily observed the emergence of a minimum in reflection, the angle of which decreases with increasing wavelength. Note that this angle can have values above (for $\lambda = 755$ nm) and below ($\lambda = 775$ nm) 45 degrees, a clear signature of the generalized Brewster's effect. We also include, for completeness, the absorption, both under s- and p-polarized incidence, in Supplementary Figs. 9d and e, respectively. It allows tracking the resonances excited in the system. For p-polarized light, comparison with Supplementary Figs. 9a and b also serves as a verification of the resonances computed by the multipole decomposition.

Supplementary Note 8: Radiation patterns of each nanodisk in a square lattice on top of fused silica substrate leading to vanishing reflection at p-polarized incidence.

In this section, the radiation patterns emitted by pairs of electric and magnetic dipoles excited in each silicon nanodisk in the array retrieved through the multipole decomposition are presented. Parameters of the simulated array are the same as in Fig.5 from the main text (disk diameter $D = 170$ nm, disk height $H = 160$ nm, array pitch $P = 300$ nm and substrate refractive index is of 1.45). Supplementary Figs. 10a and b show the radiation patterns of the dipoles at wavelengths of 590 nm and 735 nm leading, respectively, to a minimum reflection at 25° and 60° of incidence, respectively. The radiation patterns were computed with Stratton-Chu equations[5] taking into account the presence of the substrate. For these calculations, a sphere enclosing the dipoles and the substrate was considered. Although the solution is not exact, convergence against variations in the radius of the sphere was checked, yielding almost the same results. Both radiation patterns show minima in the direction of the reflected wave, thus confirming the interference origin of the observed vanishing reflection effect also in the case of silicon disks on substrate.

Supplementary Note 9: Explicit expressions used in the Multipole Decomposition.

Multipole decomposition technique was employed to analyze the different modes excited inside the particles. For particles in an array embedded in air, multipoles can be computed through the polarization currents induced inside them:

$$\mathbf{J} = -i\omega\varepsilon_0(\varepsilon - 1)\mathbf{E}, \tag{17}$$

where ε is the permittivity of the particle and $\mathbf{E} = \mathbf{E}(\mathbf{r})$ the electric field inside it.

This approach fully takes into account mutual interactions in the lattice[6] as well as the possible presence of a substrate. Cartesian basis with origin in the center of the particles was used in the present work. An accurate description of the radiative properties in this basis involves the introduction of the family of toroidal moments[7] and the mean-square radii corrections. Although the explicit expression of the multipoles can be found in some references (see, in particular Ref.7, for the explicit connection with the usual spherical multipole moments) we repeat them here for completeness.

The dipolar moments induced in the system read as:

$$\mathbf{p_{car}} = \int \varepsilon_0(\varepsilon - 1)\mathbf{E}dr \tag{18}$$

$$\mathbf{m_{car}} = \frac{-i\omega}{2}\int \varepsilon_0(\varepsilon - 1)[\mathbf{r} \times \mathbf{E}]dr \tag{19}$$

$$\mathbf{t} = \frac{-i\omega}{10}\int \varepsilon_0(\varepsilon - 1)[(\mathbf{r} \cdot \mathbf{E})\mathbf{r} - 2r^2\mathbf{E}]dr \tag{20}$$

and the mean-square radii of the dipole distributions as:

$$\overline{\mathbf{R_m^2}} = \frac{-i\omega}{2}\int \varepsilon_0(\varepsilon - 1)[\mathbf{r} \times \mathbf{E}]r^2dr \tag{21}$$

$$\overline{\mathbf{R_t^2}} = \frac{-i\omega}{28}\int \varepsilon_0(\varepsilon - 1)[3r^2\mathbf{E} - 2(\mathbf{r} \cdot \mathbf{E})\mathbf{r}]r^2dr \tag{22}$$

where only the magnetic and toroidal components are considered, since the electric one does not contribute to radiation[7]. For the quadrupolar moments we have the following expressions:

$$\overline{\overline{\mathbf{Q}}}_{\mathrm{e}} = \frac{1}{2}\int \varepsilon_0(\varepsilon - 1)\left[\mathbf{r}\otimes\mathbf{E} + \mathbf{E}\otimes\mathbf{r} - \frac{2}{3}(\mathbf{r}\cdot\mathbf{E})\overline{\overline{\mathbf{I}}}\right]dr \tag{23}$$

$$\overline{\overline{\mathbf{Q}}}_{\mathrm{m}} = \frac{-i\omega}{3}\int \varepsilon_0(\varepsilon - 1)[\mathbf{r}\otimes(\mathbf{r}\times\mathbf{E}) + (\mathbf{r}\times\mathbf{E})\otimes\mathbf{r}]dr \tag{24}$$

$$\overline{\overline{\mathbf{Q}}}_{\mathrm{t}} = \frac{-i\omega}{28}\int \varepsilon_0(\varepsilon - 1)\left[4(\mathbf{r}\cdot\mathbf{E})\mathbf{r}\otimes\mathbf{r} - 5r^2(\mathbf{r}\otimes\mathbf{E} + \mathbf{E}\otimes\mathbf{r}) + 2r^2(\mathbf{r}\cdot\mathbf{E})\overline{\overline{\mathbf{I}}}\right]dr \tag{25}$$

with ⊗ being the dyadic product. It can be shown that both the Cartesian electric dipole and the toroidal dipole have the same radiation pattern. Thus, when using equation (1), the following identifications were made:

$$\mathbf{p} = \mathbf{p_{car}} + \frac{ik_0}{c}\left(\mathbf{t} + \frac{k_0^2}{10}\ \overline{\mathbf{R_t^2}}\right) \tag{26}$$

$$\mathbf{m} = \mathbf{m_{car}} - k_0^2\overline{\mathbf{R_m^2}} \tag{27}$$

The scattering cross sections in SI units then read:

$$C_{\mathrm{sca}}{}^{(\mathrm{ED})} = \frac{k_0^4}{6\pi\varepsilon_0^2E_0^2}\left|\mathbf{p_{car}} + \frac{ik_0}{c}\left(\mathbf{t} + \frac{k_0^2}{10}\ \overline{\mathbf{R_t^2}}\right)\right|^2 \tag{28}$$

$$C_{\mathrm{sca}}{}^{(\mathrm{MD})} = \frac{\eta_0^2k_0^4}{6\pi E_0^2}\left|\mathbf{m_{car}} - k_0^2\overline{\mathbf{R_m^2}}\right|^2 \tag{29}$$

$$C_{\mathrm{sca}}{}^{(\mathrm{EQ})} \approx \frac{k_0^6}{80\pi\varepsilon_0^2E_0^2}\left|\overline{\overline{\mathbf{Q}}}_{\mathrm{e}} + \frac{ik_0}{c}\overline{\overline{\mathbf{Q}}}_{\mathrm{t}}\right|^2 \tag{30}$$

$$C_{\mathrm{sca}}{}^{(\mathrm{MQ})} \approx \frac{\eta_0^2k_0^6}{80\pi E_0^2}\left|\overline{\overline{\mathbf{Q}}}_{\mathrm{m}}\right|^2 \tag{31}$$

Supplementary Note 10: Experimental setup scheme.

For completeness we present in Supplementary Figure 11 a schematic representation of the home-built experimental setup used for measuring the angular transmission/reflection from the fabricated silicon nanodisks arrays. The state of polarization of incident light is described in each of the major steps in the setup. The lamp is used for alignment purposes only and is switch-off during the measurements.

Supplementary References:

1. Landau, L. D., Lifshitz, E. M., and Pitaevsky, L. P. *Electrodynamics of Continuous Media*, Butterworth-Heinemann, Oxford, UK (2002), §86 and Problem 4.
2. Landau, L. D., Lifshitz, E. M. *The Classical Theory of Fields*, Butterworth-Heinemann, Oxford, UK (2013).
3. Bohren, C. F. & Huffmann, D. R. *Absorption and Scattering of Light by Small Particles*. A Wiley-Interscience Publication (1983).
4. Palik, E. D. & Bennett, J. M. (eds.) *Handbook of Optical Constants of Solids*. The Optical Society of America (1995).
5. Stratton, J. A. *Electromagnetic Theory*, McGraw-Hill, NY (1941).
6. Grahn, P., Shevchenko, A. & Kaivola, M. Electromagnetic multipole theory for optical nanomaterials. *New J. Phys.* **14**, 093033 (2012).
7. Radescu, E. E. & Vaman, G. Exact calculation of the angular momentum loss, recoil force, and radiation intensity for an arbitrary source in terms of the electric, magnetic, and toroid multipoles. *Phys. Rev. E* **65**, 046609 (2002).
